# Solid-Phase Quasi-Intramolecular Redox Reaction of [Ag(NH_3_)_2_]MnO_4_: An Easy Way to Prepare Pure AgMnO_2_

**DOI:** 10.1021/acs.inorgchem.0c03498

**Published:** 2021-03-01

**Authors:** Lara A. Fogaca, Éva Kováts, Gergely Németh, Katalin Kamarás, Kende A. Béres, Péter Németh, Vladimir Petruševski, Laura Bereczki, Berta Barta Holló, István E. Sajó, Szilvia Klébert, Attila Farkas, Imre M. Szilágyi, László Kótai

**Affiliations:** † Department of Inorganic and Analytical Chemistry, Budapest University of Technology and Economics, Müegyetem rakpart 3, Budapest H-1111, Hungary; ‡ Institute of Materials and Environmental Chemistry, Research Centre for Natural Sciences, Magyar Tudósok krt 2, Budapest H-1117, Hungary; § Wigner Research Centre for Physics (RCP), Institute for Solid State Physics and Optics, Konkoly Thege u. 29−33, Budapest H-1121, Hungary; ⊥ Department of Earth and Environmental Sciences, University of Pannonia, Egyetem út 10, Veszprém H-8200, Hungary; ∥ Faculty of Natural Sciences and Mathematics, Ss. Cyril and Methodius University, Skopje 1000, Macedonia; ◆ Chemical Crystallography Research Laboratory, Research Centre for Natural Sciences, University of Novi Sad, Novi Sad 21000, Serbia; ○ Department of Chemistry, Biochemistry and Environmental Protection, Faculty of Sciences, University of Novi Sad, Trg Dositeja Obradovića 3, Novi Sad 21000, Serbia; # János Szentágothai Research Centre, University of Pécs, Ifjúság útja 20, Pécs H-7624, Hungary; ¶ Department of Organic Chemistry, Budapest University of Technology and Economics, Budapest H-1111, Hungary; ∇ Deuton-X Ltd., Selmeci u2. 89, Érd H-2030, Hungary

## Abstract

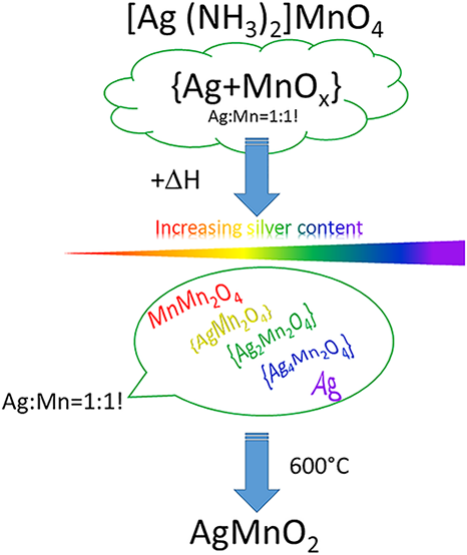

Two monoclinic polymorphs of [Ag(NH_3_)_2_]MnO_4_ containing a unique coordination mode of permanganate ions were prepared, and the high-temperature polymorph was used as a precursor to synthesize pure AgMnO_2_. The hydrogen bonds between the permanganate ions and the hydrogen atoms of ammonia were detected by IR spectroscopy and single-crystal X-ray diffraction. Under thermal decomposition, these hydrogen bonds induced a solid-phase quasi-intramolecular redox reaction between the [Ag(NH_3_)_2_]^+^ cation and MnO_4_^*–*^ anion even before losing the ammonia ligand or permanganate oxygen atom. The polymorphs decomposed into finely dispersed elementary silver, amorphous MnO_*x*_ compounds, and H_2_O, N_2_ and NO gases. Annealing the primary decomposition product at 573 K, the metallic silver reacted with the manganese oxides and resulted in the formation of amorphous silver manganese oxides, which started to crystallize only at 773 K and completely transformed into AgMnO_2_ at 873 K.

## Abstract 2

[Ag(NH_3_)_2_]MnO_4_ polymorphs containing silver cations, coordinated by two permanganate anions and alternating Ag(NH_3_)_2_^+^ units, are prepared. The hydrogen bonds between the permanganate ions and the ammonia hydrogen atoms give rise to a solid-phase quasi-intramolecular redox reaction between the [Ag(NH_3_)_2_]^+^ and MnO_4_^−^ ions upon heating before losing the ammonia ligand. The finely dispersed silver and amorphous manganese oxides formed at 300 °C result in amorphous silver manganese oxides, which crystallize at 600 °C into AgMnO_2_.

## Introduction

Solid-phase quasi-intramolecular redox reactions of compounds containing redox-active cations and anions ensure an easy way to prepare nanosized transition-metal oxides, which can be used as catalysts and sensors.^1−5^ Reduction of AgMnO_4_ to {AgMnO_*x*_} (*x* = 2–3; formulas given in { } mean materials with known chemical but unknown phase compositions) type materials plays a key role in the preparation of highly efficient catalysts in CO oxidation^6^ and in the combustion of N-heterocycles and chlorinated compounds (Körbl catalysts).^7^ Because of the high reactivity of silver permanganate, however, control of the thermal decomposition process is difficult and production of the above-mentioned catalysts on a large scale has remained a serious challenge. In particular, AgMnO_2_ is a promising candidate of delafoyssite-type (CuMnO_2_) thin films and solar cell components for the preparation of high-energy-density cells. However, its reported synthesis consists of risky steps and potentially explosive reactions such as the autoignition process of AgNO_3_ and manganese nitrate with ethylene glycol.^8,9^ Therefore, the safe preparation of AgMnO_2_ in a phase-pure form without the formation of accompanying contaminants is demanding. The temperature-controlled quasi-intramolecular/intracrystal redox reaction of a high-valence manganese oxoacid silver salt looks like an easy and promising method to prepare mixed-metal oxides. Therefore, it is an interesting task to prepare complexes of AgMnO_4_ and reduce the permanganate ions with their ligands, which can act as quasi-intramolecular reducing agents at the molecular level. Using pyridine as a ligand/reducing agent results in complexes that during heating decompose into Ag/Mn_3_O_4_ composites without the formation of AgMnO_*x*_ compounds.^4,10^ Although there is no information about the reductive thermal decomposition of silver permanganate ammonia (NH_3_) complexes, other transition-metal permanganate complexes could easily be transformed into spinel-like mixed oxides (MMn_2_O_4_, where M = Cu, Zn, and Cd).^11−14^ Our previous successful work on the synthesis and studies on the decomposition of compounds having redox-active cationic/anionic parts^3,4,10^ prompted us to study ammonia complexes of silver permanganate as potential precursors in the low-temperature (<373 K) preparation of nanosized Körbl and CO oxidation catalysts.

Three ammonia complexes of silver permanganate have been described (Table 1): [Ag(NH_3_)_2_]MnO_4_ (**1**), its monohydrate (**2**), and [Ag(NH_3_)_3_]MnO_4_ (**3**). In principle, the hydrogen content of three ammonia ligands (9 H) in compound **3** is enough to complete the reduction of one permanganate (four oxygen atoms) into metallic silver and manganese. Here, our goal is the preparation of {AgMnO_*x*_} phases using the least hydrogen-rich compounds **1** and **2**. Although compound **1** has been well-known for a long time, there is no solid evidence about the existence of compound **2**. Only Scagliari and Marangoni mentioned its existence and declared it to be isomorphous with the hydrated diamminesilver(I) perchlorate (**2-Cl**).^15^

**Table 1:** Ammonia Complexes of Silver Permanganate^a^

compound	label	ref
[Ag(NH_3_)_2_]MnO_4_	**1**	(12), (23)
[Ag(NH_3_)_2_]MnO_4_·H_2_O	**2**	(15)
[Ag(NH_3_)_3_]MnO_4_	**3**	(20)
[Ag(NH_3_)_2_]MnO_4_, low-temperature polymorph	**LT-1**	present work
[Ag(NH_3_)_2_]MnO_4_, high-temperature polymorph	**HT-1**	present work

a The analogue perchlorate compounds were also prepared^15,20,21,24,25^ and marked with ClO_4_.

The structures of ammine complexes of transition-metal permanganates play a key role in initiating solid-phase redox reactions and result in mixed-metal manganese oxides;^12,14^ thus, it is essential to study the existence of possible polymorphs/hydrates of **1** and elucidate their structures and thermal properties.

## Experimental Section

***Caution!** The permanganate and perchlorate compounds are potentially explosive; thus, they had to be handled with great care.* All of the chemicals used (AgNO_3_, 25% aqueous NH_3_, NaClO_4_·H_2_O, KMnO_4_, NaMnO_4_, concentrated HCl, NaOH, 8-hydroxyquinoline, acetic acid, ammonium acetate, methanol, and oxalic acid) in chemically pure form were supplied by Deuton-X Ltd., Érd, Hungary.

Gravimetric analysis of the manganese and silver contents was performed by dissolving the samples in HClO_4_ (HCl and H_2_SO_4_ resulted in AgCl and Ag_2_SO_4_ precipitates) and reacting them with oxalic acid to prepare silver(I)- and manganese(II)-containing solutions. The silver content was removed and analyzed as AgCl (with HCl addition), whereas the manganese(II) content was determined gravimetrically as oxinate.^3,4^ The excess oxalic acid was measured with titration using 0.02 M KMnO_4_ according to the standard procedure. The ammonia content was removed with the addition of 10% NaOH, and the air/ammonia mixture was sucked out through a sulfuric acid solution using a vacuum (in order to avoid ammonia oxidation by the permanganate, we could not boil off the ammonia by heating). Finally, the sulfuric acid excess was measured back with 0.1 M NaOH in the presence of a methyl orange indicator.

Fourier transform infrared (FT-IR) spectra of crystalline samples were recorded in the attenuated-total-reflection mode on a Bruker Alpha FT-IR spectrometer (resolution: 2 cm^–1^) and on a Biorad Excalibur Series FTS 3000 IR spectrometer, in KBr pellets between 4000 and 400 cm^–1^. Far-IR measurements were registered on a BioRad-Digilab FTS-30-FIR spectrometer for the 400–40 cm^–1^ range in a Nujol mull between polyethylene plates. The low-temperature IR measurements were performed on a Bruker IFS 66v FT-IR spectrometer in KBr pellets between 400 and 4000 cm^–1^ with 2 cm^–1^ resolution in a liquid-nitrogen-cooled flow-through cryostat. Transmission electron microscopy (TEM) data were acquired with a 200 keV Talos Thermo Scientific transmission electron microscope. The grains of samples were crushed under ethanol and deposited onto copper grids covered by Lacey carbon. We obtained bright-field TEM (BFTEM), high-resolution TEM (HRTEM), and high-angle annular dark-field (HAADF) images as well as selected-area electron diffraction (SAED) patterns. The chemical composition of the grains was measured with a “Super-X” detector system having four silicon drift detectors built into the microscope column.

The elemental composition of solid solutions regarding the metal content was determined by atomic emission spectroscopy using a Spectro Genesis inductively coupled plasma optical emission simultaneous spectrometer (SPECTRO Analytical Instruments GmbH, Kleve, Germany) with axial plasma observation. Multielement standard solutions for inductively coupled plasma (Merck Chemicals GmbH, Darmstadt, Germany) were used for calibration.

Single-crystal structures of two polymorphic modifications of complex **1** were determined at 100 K (**LT-1**) and 180 K (**HT-1**) using Mo Kα radiation. The intensity data were collected on a Rigaku RAXIS-RAPID diffractometer equipped with a graphite monochromator. A numerical absorption correction was applied to the data. The atomic positions were determined by a charge-flipping method.^16^ The non-hydrogen atomic positions were refined by anisotropic full-matrix least-squares refinement.^17−,19^ Hydrogen atoms were placed in geometrically calculated positions. The **LT-1** structure was refined as a nonmerohedral twin with 0.8572(18) and 0.1428(18) contributions of the twin individuals.

## Results and Discussion

### Preparation and Properties of Diamminesilver(I) Permanganate Compounds

In the literature, we found limited information about the ammonia complexes of silver(I) permanganate.^15,20−23^ Diamminesilver(I) permanganate was prepared first by Klobb^23^ in the reaction of aqueous silver nitrate and potassium permanganate dissolved in water and saturated with ammonia at 283 K. Scagliari and Marangoni described the diamminesilver(I) permanganate monohydrate, which was prepared from the reaction of an ammoniacal silver nitrate solution and potassium permanganate.^15^ The different colors of [Ag(NH_3_)_2_](ClO_4_,MnO_4_)·H_2_O solid solutions led to the conclusion about the existence of isomorphism between compound **2** and its perchlorate analogue (compound **2-ClO_4_**). Bruni and Levi^20^ repeated Klobb’s^23^ and Scagliari’s^15^ experiments, but the products in both cases were proven to be the anhydrous compound **1**. Reacting solid silver permanganate with gaseous ammonia at 283 K in 72 h resulted in compound **3**. In order to determine the identity of the compounds formed in Scagliari’s experiments^15^ and clarify the phase relationships in the products formed by the methods used by Klobb,^23^ we repeated the known preparation methods. The prepared and previously reported compounds of the AgMnO_4_/NH_3_ system are given in Table 1.

Both Klobb’s^23^ and Scagliari’s^15^ methods resulted in the same main products that we isolated from the reaction of an aqueous solution of diamminesilver(I) nitrate^26^ and sodium permanganate upon cooling to 283 K. These products were proven to be anhydrous diamminesilver(I) permanganate (compound **1**; Figure S1). The peak intensity differences of the diffractograms between the samples could be attributed to the preferred orientation. Following Scagliari’s^15^ experiment, however, a small amount of an unidentified phase was also detected. Thus, our further studies were focused on compound **1** prepared using NaMnO_4_ and [Ag(NH_3_)_2_]NO_3_. The use of NaMnO_4_ gave a better yield of compound **1** than the experiments performed with KMnO_4_.

### Polymorphism of Compound **1**

Because Scagliari’s^15^ method resulted in the anhydrous permanganate salt, the isomorphous perchlorate and [Ag(NH_3_)_2_](ClO_4_,MnO_4_) solid solutions should also be anhydrous. Nockemann and Meyer studied the structure of anhydrous diamminesilver(I) perchlorate (**1-ClO**_**4**_) in detail,^24^ and they found the existence of two polymorphs, the orthorhombic **HT-1-ClO**_**4**_ and monoclinic (low-temperature) **LT-1-ClO**_**4**_. In contrast to Scagliari’s results, the orthorhombic room-temperature polymorph was not isomorphous with the room-temperature monoclinic form of compound **1**. This controversial result encouraged us to study the existence of other polymorphs with the composition of compound **1**. Differential scanning calorimetry (DSC) studies were performed on compounds **1** and **1-ClO**_**4**_ between 123 and 303 K. On the basis of the results (Figures S2 and S3), similar to perchlorates, the permanganate complex (compound **1**) had two monoclinic polymorphs. Only the low- temperature forms (compounds **LT-1** and **LT-1-ClO**_**4**_) were isomorphic, whereas the room-temperature forms were distinct phases (compounds **HT-1** and **HT-1-ClO**_**4**_). The peak temperature of the phase changes and enthalpy values for compounds **LT-1** and **HT-1** or **LT-1-ClO**_**4**_ and **HT-1-ClO**_**4**_ determined by DSC are given in Table 2.

**Table 2:** Phase Transition Temperatures and Enthalpies for Compounds **1** and **1-ClO**_**4**_

phase transition	*T*, K	Δ*H*,kJ/mol	ref
**LT-1** → **HT-1**	162.3	1.107	present work
**LT-1-ClO**_**4**_ → **HT-1-ClO**_**4**_	225.7	1.030	present work
	200–210	not measured	(23)

The phase transition temperature for the permanganate complex was ∼60 K lower than that of the perchlorate compound, but the enthalpy values of the phase transitions were close to each other. Because there was no isomorphism between the room-temperature forms of the permanganate (compound **HT-1**) and perchlorate (compound **HT-1-ClO**_**4**_) salts, we studied the possible reasons why they could form solid solutions with each other. The monoclinic cell of **HT-1** and the orthorhombic cell of **HT-1-ClO**_**4**_ were very similar in size (Table 5). In fact, the orthorhombic cell was a special case of the monoclinic cell with the unique angle β equivalent to 90°. In principle, two solid solutions (a monoclinic and an orthorhombic) may be expected with or without concentration limits and with variable *a*, *b*, *c*, and β parameters between compounds **HT-1** and **HT-1-ClO**_**4**_. To identify the types of the solid solutions formed, a series of reactions were prepared by continuously increasing the perchlorate/permanganate ratio (∼1:9, ∼3:7, ∼1:1, ∼7:3 and ∼9:1, 11.5:1, 13:1, 20:1, and 100:1) in the starting NaClO_4_/KMnO_4_ (NaMnO_4_) solution. The composition and crystal system of the isolated solid solutions with their starting ClO_4_^–^/MnO_4_^–^ ratios are given in Table 3.

**Table 3:** Composition and Lattice Type of the Solid Solutions Made from [Ag(NH_3_)_2_]NO_3_ and (K,Na)(MnO_4_,ClO_4_) Solutions

permanganate used	solution-phase ClO_4_/MnO_4_ ratio	ClO_4_/MnO_4_ ratio in the solid solution	crystal structure
KMnO_4_	99:1	97:3	orthorhombic
KMnO_4_	95:5	86:14	orthorhombic
KMnO_4_	92:8	72:28	orthorhombic + monoclinic
KMnO_4_	90:10	69:31	monoclinic
KMnO_4_	70:30	62:38	monoclinic
KMnO_4_	50:50	26:74	monoclinic
NaMnO_4_	30:70	23:77	monoclinic
NaMnO_4_	10:90	05:95	monoclinic

**Figure 1:**
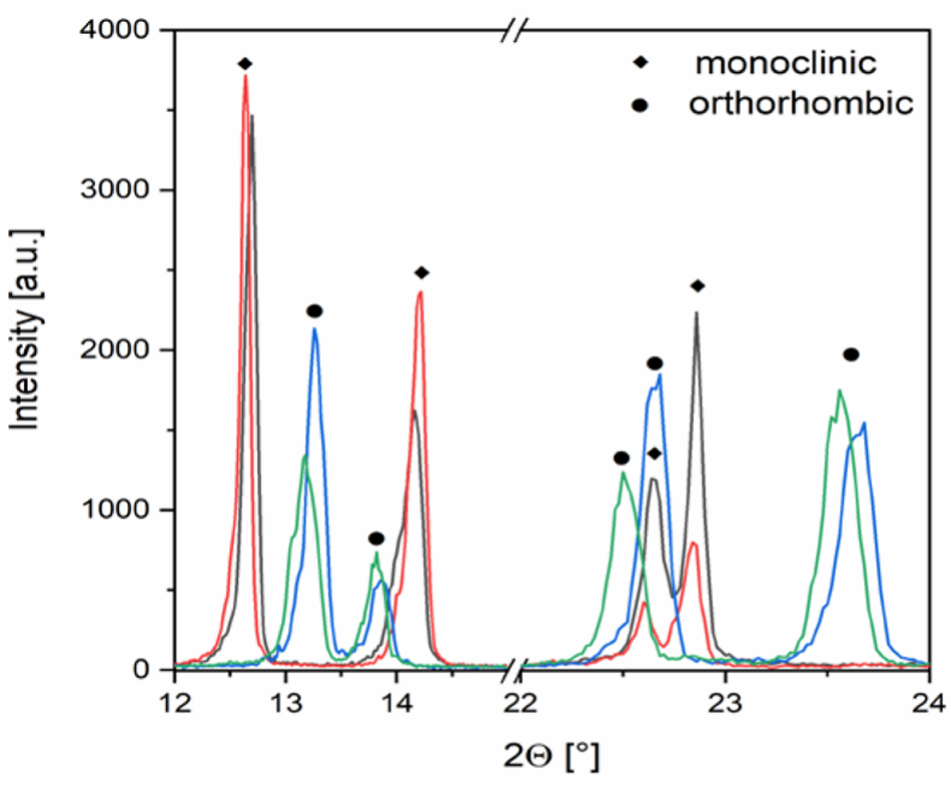
Powder XRD patterns of [Ag(NH_3_)_2_](ClO_4_,MnO_4_) solid solutions (green and blue, 3 and 14 mol % permanganate ion content, orthorhombic lattice, respectively; purple and red, 31 and 77% permanganate ion content, monoclinic, respectively).

The reaction of [Ag(NH_3_)_2_]NO_3_ with (K,Na)(MnO_4_,ClO_4_)- containing solutions with smaller than 3:7 MnO_4_^–^/ClO_4_^–^ molar ratio resulted in precipitates immediately even at room temperature. However, the solution with 1:1 MnO_4_^–^/ClO_4_^–^ molar ratio had to be cooled to obtain crystalline materials. The low solubility of KMnO_4_ required the use of as much water as could dissolve the desired product at room temperature. When the ratio of KMnO_4_/NaClO_4_ was increased, upon cooling the solutions, only KMnO_4_ was precipitated out. Therefore, the solid solution products with MnO_4_^–^/ClO_4_^–^ molar ratio greater than 3 (solution phase) could only be prepared by using highly soluble NaMnO_4_. (The solubility of NaMnO_4_ is higher with almost 1 order of magnitude than the solubility of potassium permanganate,^16^ which ensures an easier way to prepare the sparingly soluble permanganate complexes than the generally used routes.) The increase of the KMnO_4_/NaClO_4_ molar ratio in the starting reactant resulted in a continuous increase of the permanganate content in the formed solid solutions (Table 3). Two kinds of solid solutions, a monoclinic and an orthorhombic, were isolated (Figure 1). No miscibility gap was found. The phase transformation occurred with ∼28 mol % permanganate content (Table 3).

The X-ray diffraction (XRD) diffractograms of samples with 3 and 14 mol % (green and blue) and 31 and 77 mol % (purple and red) permanganate ions were consistent with those of orthorhombic HT-[Ag(NH_3_)_2_]ClO_4_ and monoclinic HT-[Ag(NH_3_)_2_]MnO_4_ (Figures S4 and S5), respectively. At ∼28 mol % permanganate ion content, both phases existed together (Figure S6). Several XRD peak positions of the orthorhombic and monoclinic solid solutions diffractograms were shifted compared to the peaks of the pure perchlorate and permanganate phases, respectively, due to differences in the size of perchlorate and permanganate ions.

**Figure 2:**
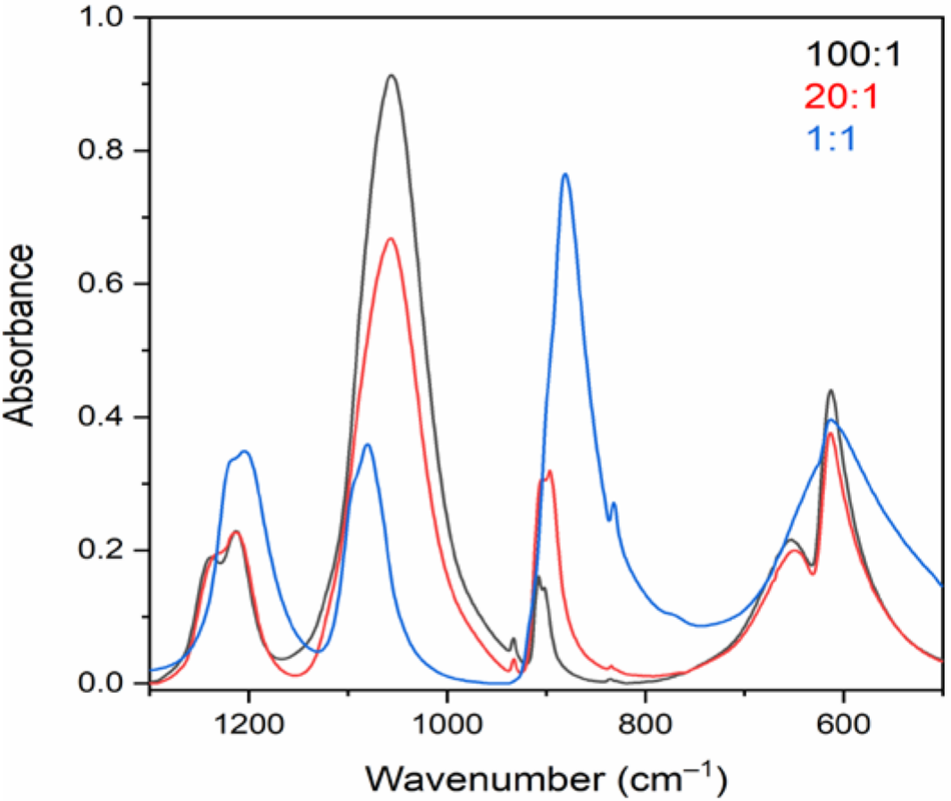
IR spectra of [Ag(NH_3_)_2_](ClO_4_,MnO_4_) solid solutions with 3 mol % (100:1), 14 mol % (20:1), and 74 mol % (1:1) permanganate content.

The IR spectra of [Ag(NH_3_)_2_](ClO_4_,MnO_4_) solid solutions with 97:3, 86:14, and 26:74 ClO_4_/MnO_4_ ratios (blue, purple, and green lines, respectively; Figure 2) unambiguously showed the gradual substitution of perchlorate and permanganate ions. The relative intensities of ν_as_(Cl–O)(F_2_) perchlorate bands at ∼1080 cm^–1^ decreased in comparison to those of ν_as_(Mn–O)(F_2_) permanganate bands at ∼900 cm^–1^. These bands also showed an appreciable shift in their peak positions, which increased with increasing permanganate concentrations in the solid solutions. The peak positions of the perchlorate and permanganate ions were shifted to higher and lower wavenumber values, respectively, with increasing permanganate concentration. The wavenumber values and relative intensities of the ν_as_(Cl–O and Mn–O)(F_2_) IR peaks measured on the solid solution samples are given in Table 4.

**Table 4:** Intensities and Positions of Asymmetric Stretching Modes of Perchlorate and Permanganate Anions in the [Ag(NH_3_)_2_](ClO_4_,MnO_4_) Solid Solutions

ClO_4_/MnO_4_ ratio	ν_as_(Cl–O)(F_2_), cm^–1^	ν_as_(Mn–O)(F_2_), cm^–1^	*I*_Cl–O_/*I*_Mn–O_
97:3 (100:1)	1054	909, 902	34.3
86:14 (100:5)	1055	908, 895	6.04
69:31 (9:1)	1071	888	2.27
62:38 (7:3)	1078	886	1.66
26:74 (1:1)	1080	884	0.34
23:77 (3:7)	1082	883	0.30
5:95 (1:9)	1094	881	0.05

### Crystallographic Characterization of Polymorphs **LT-1** and **HT-1**

We have tried to collect single-crystal XRD data for complex **1** at room temperature, but the compound always decomposed during the measurement. Therefore, the single-crystal structures of its polymorphic modifications were determined at 100 K (**LT-1**; CCDC 2044599) and 180 K (**HT-1**; CCDC 2044600). Both modifications crystallized in the monoclinic crystal system. The low-temperature modification (**LT-1**) had a lower *P*2/*m* symmetry, which was a maximal nonisomorphic symmetry subgroup of **HT-1** (*I*2/*m*). Crystal data and details of the structure determination and refinement are listed in Tables 5 and S1.

**Table 5:** Lattice Parameters of Compounds **LT-1** and **HT-1**

parameter	**LT-1**	**HT-1**
*a*, Å	7.9095(5)	7.8112(3)
*b*, Å	6.0205(4)	6.0682(2)
*c*, Å	12.6904(11)	13.1260(5)
β, deg	98.056(7)	96.4388(4)
*V*, Å^3^	598.34(8)	618.25(4)
*d*_calcd_, g/cm^3^	2.896	2.803

**Figure 3:**
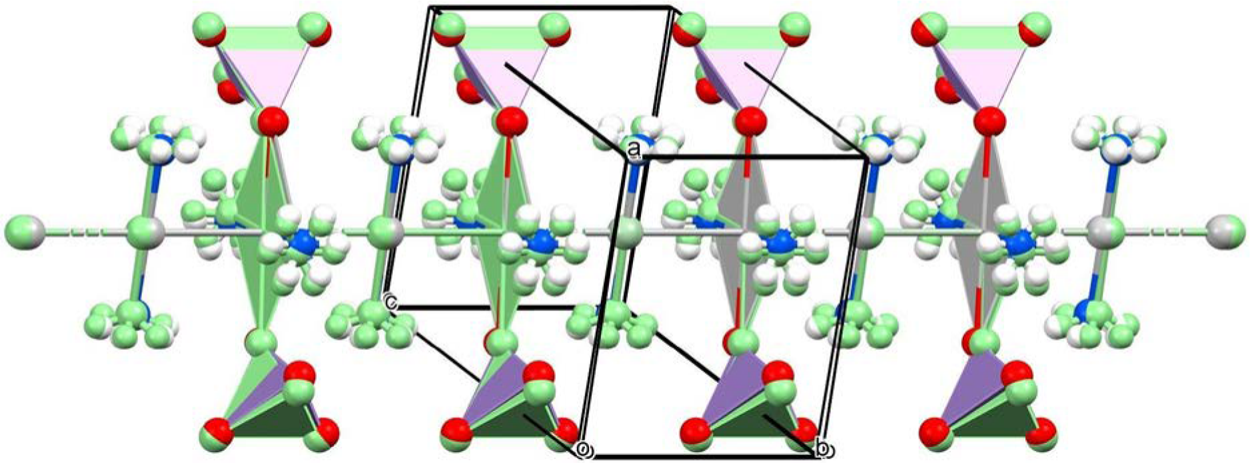
Structures of the silver chains of compounds **LT-1** (colors by elements) and **HT-1** (light green) modifications. The coordination around the silver ions alternates between square-planar (comprising two argentophilic interactions and two coordinated ammonia molecules) and octahedral (including the coordination of two additional permanganate anions).

**Figure 4:**
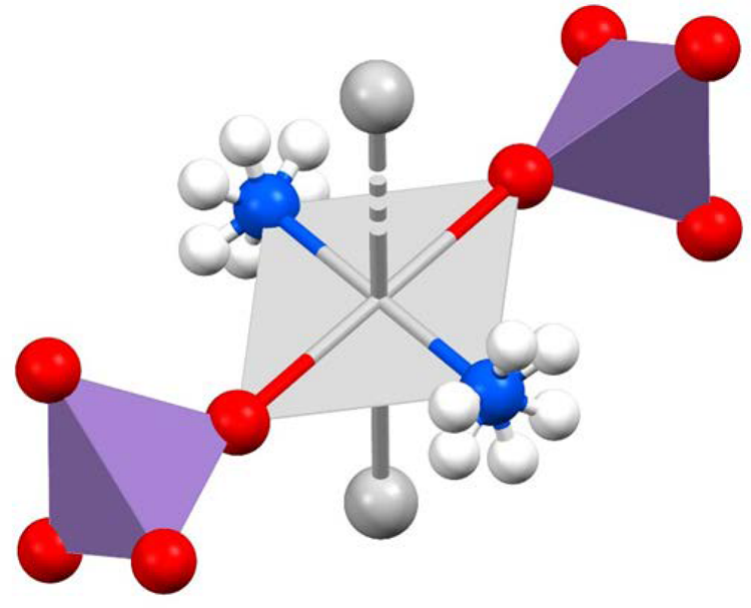
Octahedrally coordinated atoms around the silver ion.

**Figure 5:**
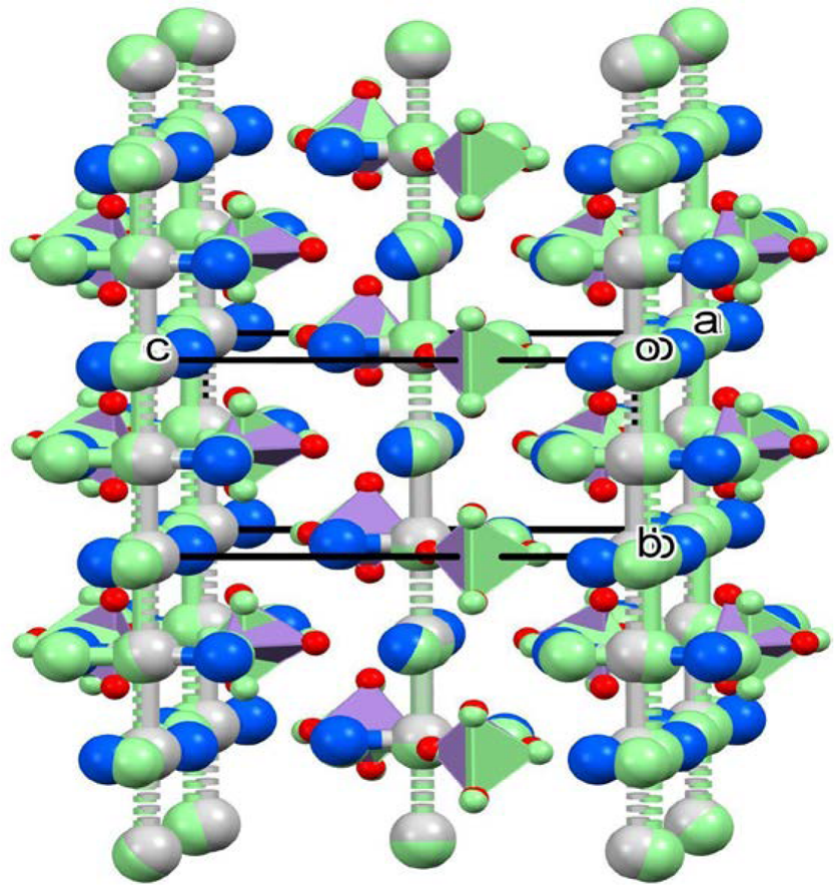
Comparison of the similar packing in the lattices of **LT-1** (colors by elements) and **HT-1** (light green) modifications.

**LT-1** was isomorphous with the known structure of the **LT-1-ClO**_**4**_ complex, but **HT-1** was distinct from **HT-1-ClO_4_**. The asymmetric unit of **LT-1** contained four quarter silver(I) cations, four halves of ammonia ligands, and two halves of permanganate anions. In contrast, the contents of the asymmetric unit of **HT-1** were half of the atoms of **LT-1** because of its higher symmetry. The unit cells and structural motifs of the two modifications were quite similar (Table 5 and Figure 3), but the symmetry relationships of the asymmetric units as well as the bond distances and angles were different. The two permanganate anions were coordinated by every second silver ion in both structures, giving rise to a unique three-dimensional coordination network (Figures 5 and S7). The coordination geometry around every second silver ion is octahedral via coordination of the two neighboring silver ions (argentophilic interactions), two permanganates, and two ammonia molecules. The axial “neighbor” silver ions have SP-4 geometry based on the two octahedrally coordinated silver ions (argentophilic interaction) and two ammonia molecules. All of the ammonia ligands, coordinated permanganates, and “argentophilic”-bonded silver ions are in trans arrangements. The (O,O)Ag^OC-6^(Ag^SP-4^,Ag^SP-4^)(N,N) octahedra (O and N represent the coordinated permanganate and ammonia, respectively) are much more distorted in the structure of **HT-1** than in that of **LT-1** (Table S2). Every silver cation has two trans-coordinated ammonia molecules, and the hydrogen atoms of the ammonia molecules are disordered over two positions in both structures through a mirror plane (Figures 4 and S8).

Silver ions formed infinite chains parallel to the *b* crystallographic axis in both structures (Figure 3). The Ag–Ag distance is half of the length of the *b* crystallographic axes of **LT-1** (3.010 Å) and **HT-1** (3.034 Å), which were very close to those found in low- and high-temperature modifications of the analogous perchlorate complexes (3.020 and 3.089 Å for compounds **LT-1-ClO_4_** and **HT-1-ClO_4_** at 170 and 293 K, respectively). The Ag–Ag chains coincided with the 2-fold rotation axes, the Ag–N bonds are on mirror planes, and the silver ions sit on the inversion centers in both structures. All of the permanganate anions were cut in half by mirror planes. In the high-temperature modification parallel to the 2-fold rotation axes, 2-fold screw axes linked the Ag–Ag chains to each other. Besides, between every two mirror planes, a glide plane exists, which maps the silver coordination spheres to each other. Thus, the permanganate anions are related by the inversion centers.

In the **HT-1** structure, the Ag–N bonds are on the *a* and *c* unit cell axes, whereas in **LT-1**, the Ag–N bonds are tilted from the unit cell axis directions. The Ag–N distances in compounds **LT-1** and **HT-1** (2.100–2.150 and 2.112–2.113 Å, respectively) are consistent with the range found for various [Ag(NH_3_)_2_]X-type compounds (2.110–2.160 Å, Table S3). The Ag(NH_3_)_2_ units are turned by 74.75/83.16 and 82.02° (ladderlike structure) in the **LT-1** and **HT-1** polymorphs, respectively (Figure S9a,b). Significant differences in the Ag–O distances can be found in compounds **LT-1** and **HT-1**. The planes of the nitrogen and oxygen atoms are perpendicular to the silver chains in both compounds; the N–Ag–O angles are listed in Table S2.

All of the permanganate oxygen atoms are involved in the formation of hydrogen bonds with the ammonia hydrogen atoms. The hydrogen-bond parameters for **LT-1** and **HT-1** polymorphs are listed in Table S4. In the solid phase of **LT-1** and **HT-1**, an extensive hydrogen-bonded network exists with the participation of ammonia molecules and permanganate oxygen atoms. The ammonia hydrogen atoms were disordered between two positions, which, in fact, coincided with the two different hydrogen-bonding positions of the permanganate anions (white and light-blue hydrogen atoms, Figure S8). This observation suggests a certain flexibility for the ammonia positions with switching between two hydrogen-bonding sites.

The packing in the crystal lattice is similar in the **LT-1** and **HT-1** compounds, and their comparison can be seen in Figure 5. The Kitaigorodskii parameters (the ratio of the molecular volumes and unit cell volume) were found to be 81.1 and 79.6% for compounds **LT-1** and **HT-1**, respectively. The cell similarity indices for the **LT-1** and **HT-1** compounds and **LT-1** and **LT-1-ClO**_**4**_ were found to be 0.01446 and 0.01264, respectively.^27^

According to the above-mentioned structural motifs, there were four and two crystallographically different permanganate environments in the **LT-1** and **LT-2** compounds, respectively.

### IR and Raman Spectroscopic Features of Compound **1** Polymorphs (Compounds **LT-1** and **HT-1**)

In order to understand the IR and Raman spectroscopic data, we performed factor group analyses for both low-temperature (**LT-1**) and high-temperature (**HT-1**) polymorphs based on the corresponding space groups. As a result of this analysis, we could demonstrate how the originally isolated, tetrahedral modes transform under the given site symmetry/factor group (more precisely, the unit cell group) symmetry and could be properly assigned to the [Ag(NH_3_)_2_]^+^ cation and permanganate anion vibrational modes. The temperature-dependent IR and Raman spectral data are given in Figures S10–S12 and Tables 6 and 7, respectively. The far-IR spectra (Figure S13), however, could be registered only at room temperature. The Raman counterparts of the cation modes were found to be too weak in the spectra of solid complexes; thus, only the Raman bands belonging to permanganate ions could be assigned (Figure S12) in the Raman spectra recorded at 123 K (compound **LT-1**) and 183 K (compound **HT-1**), above and below ∼162 K, the temperature of the phase transition of compound **1**. When the temperature is increased , compound **HT-1** rapidly decomposes under 532 nm laser illumination, and only the bands belonging to the formed manganese oxides appeared (Figure S14).

**Table 6:** IR and Raman Data for Permanganate Ions Located in Compounds **LT-1** and **HT-1**

	compound **HT-1**			compound **4** (**LT-1**)	
assignation	IR (298 K)	Raman (183 K)	IR (180 K)	IR (87 K)	Raman (123 K)
ν_s_(A_1_)	832	833	844,830	849,829	831
δ_s_(E)	344 (wide)	346			345
ν_as_(F_2_)	900, 884, 879	907, 898	911, 893, 884	912, 893, 884	905, 897
δ_as_(F_2_)	375 (wide)	391			390

**Table 7:** IR Spectra of the Cation Part in Compounds **LT-1** (87 K) and **HT-1** at 180 and 300 K^a^

	compound **LT-1**	compound **HT-1**	
assignation	87 K	180 K	300 K
ν_as_(NH_3_) (A_1_)	3348sh	3340sh, 3314	3314
	3339	3304, 3293sh	
	3331		
	3319sh		
	3312		
	3299		
	3292		
2 × δ_as_(NH_3_)	3267	3257	3247
	3255sh	3234	3234
	3247		
	3232		
ν_s_(NH_3_) (A_1_)	3203	3184	3187
	3187	3154	3150
	3179		
	3165sh		
	3153		
δ_as_(NH_3_) (E)	1605	1602	1589
	1591	1586	
	1588		
	1580		
δ_s_(NH_3_) (E)	1231sh	1225	1222sh, 1183
	1221	1189	1171sh
	1192	1182	
	1186	1173, 1157sh	
	1180		
	1173		
ρ(NH_3_)	675sh	669sh	612
	646	640	569
	629	620	
	623sh	598	
	605sh	572	
	583	530	
	574		
	570		
	546		
	535		
	529		
ν_s_(AgN)	404,400	403, 400	404
ν_as_(AgN)	456	451	not detectable
δ(NAgN,OAgN)	∼200		

a sh = shoulder.

On the basis of the correlation analysis for polymorphs **LT-1** and **HT-1** (Figures S15–S19), nine internal modes of the permanganate ion can be expected under the *C*_*s*_ site symmetry and *C*_2*h*_ factor groups (ν_s_, δ_s_, 2ν_as_, 2δ_as_) (A_g_ and B_u_) and (δ_s_, ν_as_, δ_as_) (A_u_ and B_g_), and all of them are IR- and Raman-active. Compound **LT-1** has two different permanganate ion types; thus, the number of vibrations is twice as large as that of compound **HT-1**. Because of the external MnO_4_^–^ vibrations (hindered translations and hindered rotations), the total number of factor-group modes is equivalent to 2 × 12 = 24 and has 12 external vibrational degrees of freedom (Figure S15) for compounds **LT-1** and **HT-1**, respectively. The modes assigned to the permanganate ion in compounds **LT-1** and **HT-1** are given in Table 6.

The complex cation [Ag(NH_3_)_2_^+^] modes decomposed into components of ammonia as the ligand (*C*_3*v*_) modes and to the translation of central silver ions. The total numbers of factor group modes, due to the internal vibrations and four or two types of crystallographically different ammonia ligands, are 4 × 12 = 48 and 2 × 12 = 24, resulting in 48 and 24 vibrational degrees of freedom in compounds **LT-1** and **HT-1**, respectively. The external modes (*T*_*xy*_ and *R*_*xy*_) are doubly degenerate modes under *C*_3*v*_. The total numbers of factor-group modes, due to the external vibrations, are doubled and quadrupled (a consequence of two and four crystallographic types of NH_3_) and are equal to 4 × 12 = 48 and 2 × 12 = 24 vibrational degrees of freedom for compounds **LT-1** and **HT-1**, respectively. Regarding the Ag^+^ ions, there are 3 modes of acoustic origin, out of the total of 48 (compound **LT-1**) and 24 (compound **HT-1**) external modes, which belong to species A_u_ + 2B_u_. A total of 45 and 21 optical modes of translational origin, 72 and 36 optical modes of rotational origin, and 84 and 42 optical modes due to internal vibrations for compounds **LT-1** and **HT-1**, respectively.

### Assignation of Vibrational Modes in Polymorphs **LT-1** and **HT-1**

Two modes of the permanganate ion (δ_s_ and δ_as_) appeared only in the far-IR range. The two singlet Raman bands belonging to the ν_s_(Mn–O) modes at 833 cm^–1^ (compound **LT-1**) and 831 cm^–1^ (compound **HT-1**) were the most intense Raman bands of these compounds. The ν_as_(Mn–O) bands around 900 cm^–1^ were split into doublets at 123 K (compound **LT-1**) and 183 K (compound **HT-1**), which can be attributed to the presence of two crystallographically different permanganate positions (compound **LT-1**) or distortion of the permanganate ion symmetry.

The IR spectra of compounds **LT-1** (87 K) and **HT-1** (180 K) showed the appearance of two very weak singlet bands of the ν_s_(Mn–O) (A_1_) mode (Table 6). The ν_s_ band became a singlet at room temperature in the IR spectrum of compound **HT-1** (Table 6 and Figure S11). The appearance of the ν_s_ and δ_s_ modes shows the symmetry reduction of the permanganate ion, and the two ν_s_ bands confirm the presence of two crystallographically different permanganate sites in compound **LT-1**. The intensity ratio of ν_s_(Mn–O)/ν_as_(Mn–O) in the IR spectra of compounds **LT-1** and **HT-1** was opposite to the intensity ratio of these bands found in the Raman spectra (Figure S12). Accordingly, the ν_as_ band intensity in the IR spectrum of compounds **LT-1** and **HT-1** was the highest.

### Cation Modes

**Figure 6:**
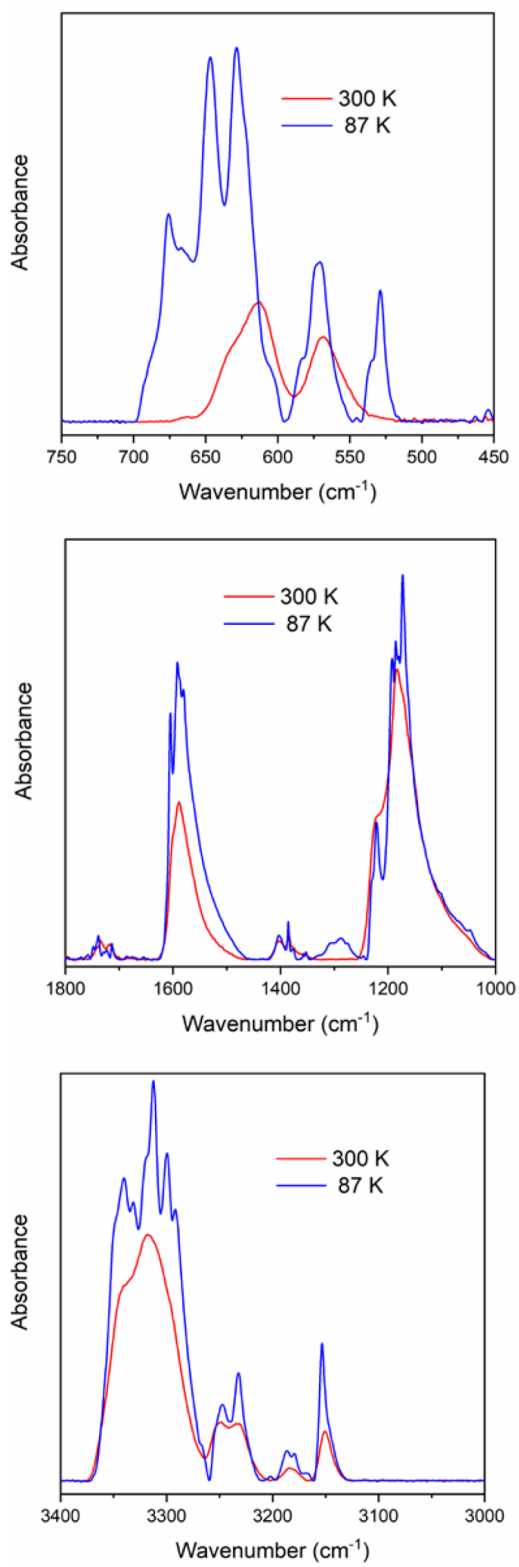
Comparison of the IR spectra of compound **1** polymorphs (87 K, compound **LT-1**; 300 K, compound **HT-1**).

The Ag–N and NH_3_ modes of the [Ag(NH_3_)_2_]^+^ cation were assigned according to the modes found in the IR and Raman spectra of [Ag(NH_3_)_2_]X compounds, where X is nitrate, sulfate, or perchlorate^24,28−32^ (Table 7). The Raman spectra of solid compounds did not show evaluable shifts for cationic modes. The temperature dependence of the IR spectroscopic parameters gave rise to splitting of the NH_3_ modes, which can be attributed to variation in the strength of hydrogen bonds, i.e., the rotational freedom of ammonia with increasing temperature. This splitting was more pronounced for compound **LT-1** than for compound **HT-1**. The effect of the phase change and temperature on the IR and Raman spectra of compounds **LT-1**/**HT-1** can be seen in Figures 6 and S12.

A complex band system was found for compound **LT-1** at 87 K belonging to the ν_as_(NH_3_) (A_1_) modes located between 3348 and 3292 cm^–1^. The band structure did not change with increasing temperature until 160 K. Compound **HT-1** had four antisymmetric NH_3_ stretching mode components at 180 K (Table 7). Above 250 K, the ν_as_(NH_3_) band components collapsed into one wider band with a shoulder. The analogous diamminesilver(I) sulfate was characterized by freezing the rotational freedom of the ammonia ligand around ∼250 K.^33^

The bands belonging to the symmetric NH_3_ stretching mode of compounds **LT-1** and **HT-1** [ν_s_(NH_3_) 3203–3153 and 3184–3154 cm^–1^ at 87 and 180 K, respectively] were shifted compared to that with the appropriate value of the gaseous ammonia [ν_s_(NH_3_) 3337 cm^–1^]. The shift of the gaseous ammonia ν_as_(NH_3_) and ν_s_(NH_3_) wavenumbers (3414 and 3337 cm^–1^, respectively) to lower wavenumber values can be attributed to the formation of a Ag–N dative bond, and the increasing strength (covalent character) of this bond increased the magnitude of the shift.^29,30^

The antisymmetric deformation mode of compounds **LT-1** and **HT-1**, δ_as_(NH_3_) (E), resulted in four and two components of the IR spectra at 87 and 180 K, respectively. Accordingly, two (one) doublets belonging to two and one types of [Ag(NH_3_)_2_]^+^ cations were found in the lattices of compounds **LT-1** and **HT-1**, respectively. The first overtones of the antisymmetric ammine deformation mode [2 × δ_as_(NH_3_)] appeared in the NH_3_ stretching range. The band systems belonging to the symmetric NH_3_ deformation mode for compounds **LT-1** and **HT-1** were located at 1221–1173 cm^–1^ (87 K) and 1223–1166 cm^–1^ (180 K), respectively.^31,34^

The rocking mode of the coordinated ammonia was the most sensitive to the type of coordination environment. Accordingly, the four different ammonia ligands in the two different Ag(NH_3_)_2_^+^ cations in compound **LT-1** gave a complex band system in the 675–529 cm^–1^ range consisting of 11 bands. The six bands of **HT-1** found at 180 K transformed into two bands at 300 K.

The modes of Ag–N linkage in the IR spectra of compounds **LT-1** and **HT-1**, excluding the symmetric and antisymmetric Ag–N stretching modes (404 and 456 cm^–1^ for compound **LT-1** and 404 and 451 cm^–1^ for compound **HT-1**), appeared only in the far-IR region. The far-IR spectra could be recorded only at room temperature (**HT-1**, Figure S13). The wide band system centered at ∼160 cm^–1^ with the asymmetric shape (located between 210 and 90 cm^–1^) contained the NAgN bending modes at ∼200 cm^–1^, the L_1_ lattice vibration (∼116 cm^–1^), and the OAgN modes as well as the combination and overtone bands of the lattice vibrations.^29^

### Contribution of Hydrogen Bonds to the Relative Bond Strength (RBS) in Polymorphs **LT-1** and **HT-1**

The δ_s_(NH_3_) wavenumbers of metal ammonia complexes depend on the strength of the M–NH_3_ bond. Grinberg^34^ defined a linear scale of RBS in ammine complexes.^34^ The calculated RBS values in [Ag(NH_3_)_2_]^+^ cations of compounds **LT-1** and **HT-1** at various temperatures are given in Table S5. The highest RBS values were found to be 66.9 for compound **LT-1** at 87 K and 65.5 and 64.8 for compound **HT-1** at 180 and 300 K, respectively. These values correspond to the 20.6, 19.1, and 18.2% contribution of hydrogen-bond interactions for the strongest hydrogen-bond position. The temperature and phase transformation had only a small influence on the maximal value of the RBS parameter according to the single-crystal XRD results, which showed minor changes with the Ag–N distance and types/positions of the hydrogen bonds during the phase transformation. The finding, in agreement with Svatos and co-workers,^30,31^ suggested a much higher contribution of the Ag–N bond upon a shift of the ν_s_(NH) and δ_s_(NH) values in ammonia complexes than that of the hydrogen bonds of these ammonia ligands. The similar RBS values of the diamminesilver(I) sulfate, nitrate, and perchlorate complexes at room temperature were found to be between 53 and 68%, between 62 and 63%, and 70%, respectively. These values were comparable with those found for compounds **LT-1** and **HT-1**.

### UV Spectroscopic Results

The UV diffuse-reflectance spectrum (Figure S18) of the solid solution of compound **HT-1** (1%) in **HT-1-ClO**_**4**_ showed a wide band system, which does not allow unambiguous assignations.

### Thermal Decomposition Features of Compound **1** (**LT-1**) in the Solid Phase

Compound **1** (**HT-1**) decomposes during a highly exothermic reaction in an inert and oxidative atmosphere. The reaction proceeded at ∼354 K in both atmospheres (Figure S21, which suggested that the aerial oxygen did not play a role in initiating the decomposition process). The total mass loss was 26.7% in an inert atmosphere, which corresponded to the formation of {AgMnO_2_} with the formal release of two NH_3_ and one O_2_ (theoretical mass loss = 26.8%). The low decomposition temperature of compound **HT-1** (∼353 K) and the exothermic character of the reaction, however, strongly suggested the appearance of a heat-evolving redox process between the reducing ammonia and oxidizing permanganate anion. In an inert atmosphere, the only oxygen source was the permanganate oxygen atom.^22,28^ There was no sign of endothermic ammonia ligand loss (Figures S22 and S23). The oxidation of coordinated ammonia with silver(I) ions in the solid phase at 353 K can be ruled out because the analogous diamminesilver sulfate loses ammonia and silver sulfate forms at 473 K without the interaction of silver(I) with ammonia.^28,35^

In the decomposition reaction of **1** at 353 K, only part of ammonia is oxidized into nitrate and the decomposition is completed at 398 K (exothermic reaction, without reaction heat dissipation). The decomposition intermediate that forms at 353 K (**I-353 K**) contains the residual NH_3_ and Ag^+^ as well as NO_3_^–^. The formed nitrate anion neutralizes the charge of the silver ion. The permanganate ions do not have a role in the further (398 K) decomposition process because they completely disappear from the system even at 353 K (see below). The metallic silver forms only after the main decomposition reaction of compound **HT-1** (at 353 K) from **I-353 K** with increasing temperature to 398 K. Because **I-353 K** contains Ag^+^ and NH_3_, they can react with each other to form metallic silver.

**Figure 7:**
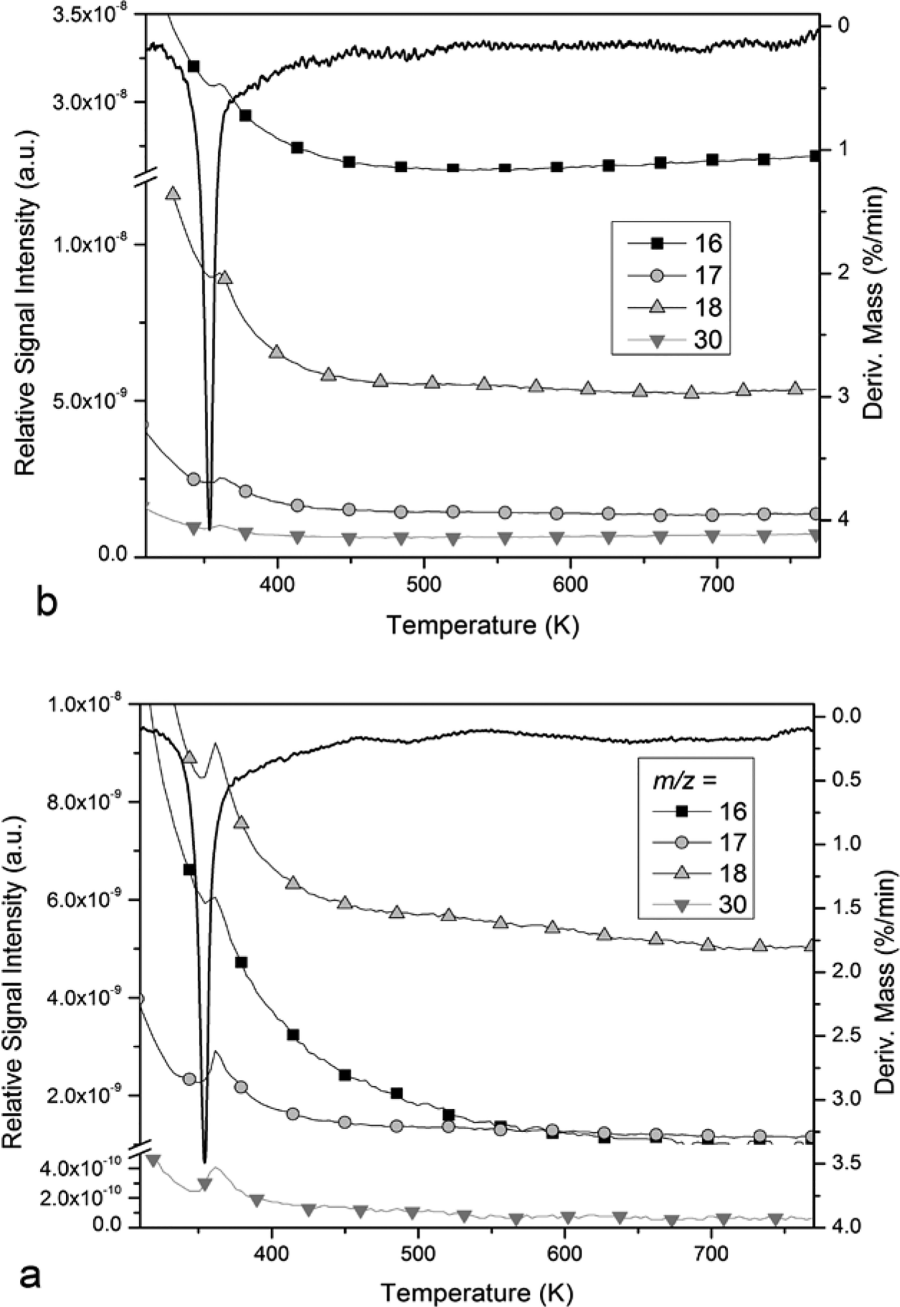
TG–MS results of compound **HT-1** under oxygen-containing air (a) and inert argon (b) atmospheres.

To better understand the decomposition mechanism of **HT-1**, we analyzed it by coupled TG–MS measurements in an inert and oxidative atmosphere. To follow the evolution of N_2_ and O_2_, the TG–MS measurements were done under argon as inert gas (Figures 7 and S27 and S28). The TG–MS data show that the main decomposition step is followed by the formation of H_2_O (*m*/*z* = 18, H_2_O^+^), N_2_ (*m*/*z* 28, N_2_^+^), NO (*m*/*z* 30, NO^+^), and N_2_O (*m*/*z* 44, N_2_O^+^) and a minor amount of O_2_ (*m*/*z* 32, O_2_^+^).

The intensity ratios of the *m*/*z* 18 (H_2_O^+^), 17 (NH_3_^+^ or OH^+^), and 16 (NH_2_^+^ and O^+^) peaks (Figure 7) confirmed that *m*/*z* 17 primarily originated from water fragmentation, and thus only a small amount of ammonia was released in the decomposition process. Similarly, we could conclude that the N^+^ fragment parent was mainly N_2_, and NO was a minor decomposition product in an argon atmosphere. (These samples could not be powdered to avoid of their decomposition during grinding.)

The reaction heat under an oxygen atmosphere was lower than that in an inert atmosphere (Δ*H* = −131.60 and −156.33 kJ/mol in O_2_ and N_2_, respectively) despite the similar character of the decomposition curves in both media (Figure S21). In an inert atmosphere, the oxygen balance was negative; therefore, only a small portion could be oxidized into NO gas, which was proven by TG–MS data (Figures 7 and S27 and S28). The main reaction could be summarized as
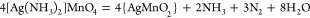
In the presence of O_2_, the oxygen balance became positive and a larger amount of NO formed than that in an inert atmosphere (Figure 7). The endothermic NO formation resulted in a decrease of the overall positive energy balance.

### Preparation and Characterization of Silver Manganese Oxides Formed by Decomposition of Compound **1**

The reaction of elementary silver or Ag_2_O with MnO, Mn_3_O_4_, and Mn_2_O_3_ in the presence of oxygen yielded various silver manganese oxides with the {Ag_*x*_MnO_2_}_*n*_ general formula like AgMnO_2_ (*x* = 1; *n* = 1), {Ag_2_MnO_2_} (*x* = 2; *n* = 1), or Hollandite-type Ag_1.8_Mn_8_O_16_ (*x* = 0.225; *n* = 8).^36−40^ Decomposition^7^ or reduction of AgMnO_4_ (1:1 Ag/Mn stoichiometry) with H_2_,^41^ H_2_O_2_,^42^ CO,^43^ or metallic silver^44^ resulted in mixed oxides with the {AgMnO_*x*_} (*x* = 2–3) composition. The composition of compound **1** and AgMnO_4_ (Ag:Mn = 1:1) predetermined the stoichiometry of the single-phase decomposition product. Multiphase products possibly contained manganese- and silver-rich phases together.

### Solvent-Mediated Decomposition of Compound **1**

The solvent-mediated temperature-limited decomposition process of metal permanganate ammine complexes was developed to prepare nanosized mixed-metal manganese oxides.^11−14^ The decomposition process was governed by the fact that the temperature of an organic solvent could not exceed its boiling point until complete evaporation of the liquid phase. Thus, using excess solvent and a reflux condenser, exothermic decomposition processes of solids could proceed smoothly in a suspension with an inert organic solvent with a boiling point close to the decomposition temperature of the particular complex. Because the decomposition temperature of compound **1** is near 353 K, benzene was an ideal oxidation-resistant solvent (bp = 353.25 K). Compound **HT-1** decomposes in a benzene suspension much slower, and decomposition proceeds in a rather controlled way compared to that in the solid phase. The aqueous extract contained only one product, identified by XRD and IR as a NH_4_NO_3_·AgNO_3_ double salt^45,46^ (Figures S24 and S25). Its formation confirmed that only part of the ammonia content oxidized into nitrate, whereas the other part remained in its original oxidation state (ammonia). The solid residue of the aqueous extraction was an almost amorphous glassy material with a ∼1.2 nm coherence distance. Metallic silver did not form in the solvent-mediated decomposition process at this temperature (353 K). Chemical analysis of the residue formed after aqueous leaching of the decomposition product prepared at 353 K gave the average composition of AgMn_2.35_O_3.83_, which indicated that ∼60 mol % of the silver was washed out as AgNO_3_·NH_4_NO_3_ and only ∼40% was incorporated into the mixed oxide phases. Assuming the presence of silver(I) in all oxide phases, the experimentally determined average charge of manganese was 2.83+.

### Solid-Phase Decomposition Intermediates of Compound **1**

**Figure 8:**
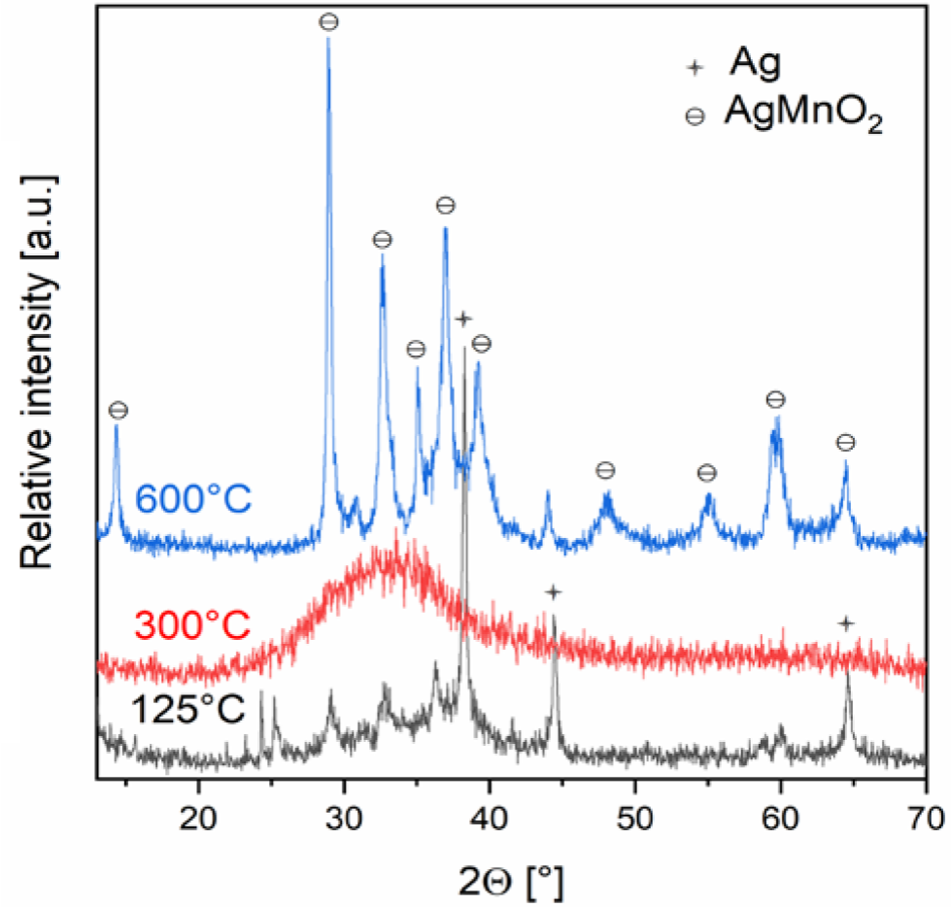
XRD of the products formed in the thermal decomposition of compound **HT-1** in air at 125, 300, and 600 °C.

In the solid phase, the enthalpy of the decomposition reaction was very high (−156.33 kJ/mol under N_2_), which resulted in local overheating and a violent reaction. In order to isolate and identify the decomposition intermediates, we investigated the products at 398 K. The XRD pattern of the mixture, however, did not contain the peaks of these compounds at all; thus, the isolable AgNO_3_·NH_4_NO_3_ could be formed only during aqueous leaching from different types of ammonium-, silver-, and nitrate-ion-containing compounds. The composition of the solid decomposition product (after aqueous leaching) was consistent with the summarized formula AgMn_3.60_O_5.74_. The decomposition product at this temperature (398 K) contained metallic silver as well as some amorphous and poorly crystallized manganese oxide phase. The finding indicated that 72% of the silver occurred as NH_4_Ag(NO_3_)_2_ and 28% was metallic silver or silver incorporated in the silver manganese oxide products. The average oxidation number of manganese was 2.60+ (Figure 8).

**Figure 9:**
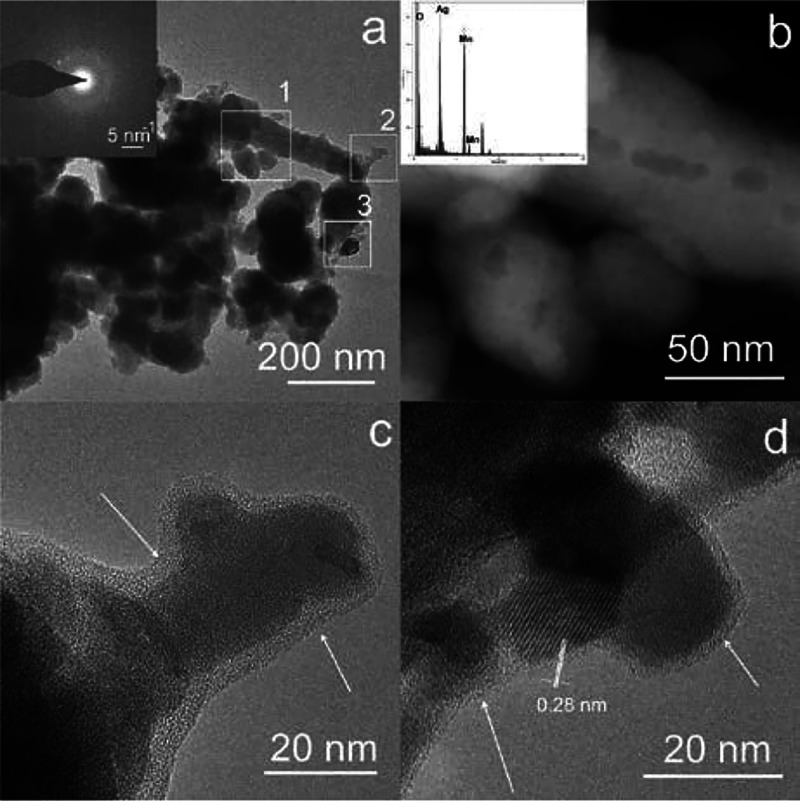
TEM images of the decomposition product heated at 300 °C. (a) Low-magnification BFTEM image and SAED pattern. (b) STEM image magnified from the area 1 marked by the white rectangle in part a and its corresponding EDS spectrum. (c) HRTEM image magnified from the area 2 marked by the white rectangle in part a. White arrows point to an amorphous shell. (d) HRTEM image magnified from the area 3 marked by the white rectangle in part a. White lines mark the 0.28 nm spacing, which presumably corresponds to the *d*{220} spacing of the spinel-like AgMn_2_O_4_.

Upon further heating, the finely divided silver easily reacted with manganese oxides (MnO, Mn_2_O_3_, and Mn_3_O_4_).^36−40^ The reaction of α-MnO_2_ does not give silver–manganese oxides, but γ- and ρ-MnO_2_ formed from the decomposition of manganese(II) nitrate^47^ react with metallic silver. The reaction, during which the elementary silver completely disappeared, proceeded in an air atmosphere even at 573 K (Figure 8). The morphology and structure of the amorphous sample formed at 573 K were studied using TEM (Figure 9). The BFTEM images showed 20–200 nm size, dominantly rounded and aggregated grains, and their corresponding SAED patterns indicated that they mainly consisted of atoms having a short-range order (Figure 9a).

According to the HRTEM images, a major part of the grains were noncrystalline and were covered by an amorphous shell (white arrows on Figure 9c). However, grains with the characteristic fringes of silver manganese oxides also occurred (Figure 9d). In fact, the diffraction pattern revealed an amorphous halo with ∼0.42 nm and some dots with 0.35 nm spacings. HAADF-STEM (also called Z-contrast) images revealed white contrast grains dotted with 1–2-nm-size gray contrast “particles” (Figure 9b). We associated these gray dots with the remnant sites of metallic silver, which presumably diffused into {Ag_*x*_MnO_2_}_*n*_.

**Figure 10:**
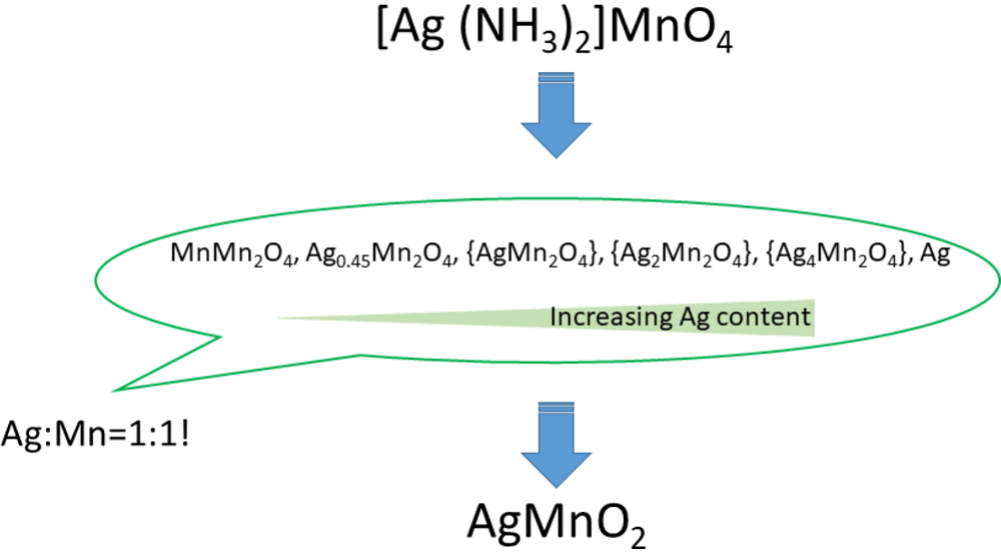
Formation of AgMnO_2_ in the decomposition of compound **1** (MnMn_2_O_4_ corresponds to Mn^II^Mn^III^_2_O_4_, Mn^III^Mn^II^Mn^III^O_4_, or their intermediates).

The amorphous and poorly crystalline material transformed into crystalline phases above 773 K, turned into phase-pure AgMnO_2_ (Figure 8) identified in refs (8) and (9) at 873 K, and decomposed above 903 K. The general scheme of the transformations of the thermal decomposition products is summarized in Figure 10. When the samples were heated up to 973 K, the product contained only ca. 20% of AgMnO_2_ and several unidentified phases possibly with the compositions of {AgMn_2_O_4_} and {Ag_2_MnO_2_} (Figure S26), which demands future studies to identify these unknown phases of the Ag–Mn–O system.

## Conclusions

Two monoclinic polymorphs of **1** have been prepared and characterized. The formerly described **2** was proven to be identical with **1**. Continuous solid solutions of orthorhombic and monoclinic [Ag(NH_3_)_2_](ClO_4_,MnO_4_) with <1 Mn/Ag stoichiometry were also synthesized. The phase transition temperature of **1** was found to be 162.3 K. A unique coordination mode between two permanganate ions and a silver cation was found in both polymorphs. The RBS values of the hydrogen bonds between the permanganate oxygen and ammonia hydrogen atoms were determined from IR measurements and single-crystal XRD studies. The hydrogen bonds acted as reaction centers to induce a solid-phase quasi-intramolecular redox reaction between the [Ag(NH_3_)_2_]^+^ cation and MnO_4_^–^ anion upon heating even before the loss of an ammonia ligand or a permanganate oxygen atom and resulted in finely divided silver and amorphous MnO_*x*_ formation. Upon annealing at 573 K, the dispersed metallic silver reacted with the manganese oxides and formed an amorphous silver manganese oxide system, which started to crystallize at 773 K and completely transformed into pure AgMnO_2_ at 873 K. The decomposition pathway of **1** was proven to be a promising new and simple way to prepare phase-pure AgMnO_2_ for potential CO oxidation and as Körbl catalyst precursors.

## Supporting Information Available

Powder XRD patterns and DSC study of compound **1** and **1-ClO**_**4**_, powder XRD patterns of **1**/**1-ClO**_**4**_ solid solutions, structures, packing information, and numerical structural data of compounds **LT-1** and **HT-1**, low- and room-temperature IR and Raman spectra and data of compounds **LT-1** and **HT-1**, far-IR and UV spectra of compound **HT-1**, correlation analysis results on compounds **LT-1** and **HT-1**, thermal analysis (TG–MS and DSC) on compound **HT-1** in various (O_2_, N_2_, Ar, and air) atmospheres, and RBS data calculated from IR (PDF)

## Accession Codes

CCDC 2044599 and 2044600 contain the supplementary crystallographic data for this paper. These data can be obtained free of charge via www.ccdc.cam.ac.uk/data_request/cif, or by emailing data_request@ccdc.cam.ac.uk, or by contacting The Cambridge Crystallographic Data Centre, 12 Union Road, Cambridge CB2 1EZ, UK; fax: +44 1223 336033.

## References

[ref1] Sajó I. E.; Bakos L. P.; Szilágyi I. M.; Lendvay G.; Magyari J.; Mohai M.; Szegedi Á.; Farkas A.; Jánosity A.; Klébert S.; Kótai L. Unexpected Sequential NH_3_/H_2_O Solid/Gas Phase Ligand Exchange and Quasi-Intramolecular Self-Protonation Yield [NH_4_Cu(OH)MoO_4_], a Photocatalyst Misidentified before as (NH_4_)_2_Cu(MoO_4_)_2_. Inorg. Chem. 2018, 57, 13679–13692. 10.1021/acs.inorgchem.8b02261. 30351069

[ref2] Kocsis T.; Magyari J.; Sajó I. E.; Pasinszki T.; Homonnay Z.; Szilágyi I. M.; Farkas A.; May Z.; Effenberger H.; Szakáll S.; Pawar R. P.; Kótai L. Evidence of quasi-intramolecular redox reactions during thermal decomposition of ammonium hydroxodisulfitoferriate(III), (NH_4_)_2_[Fe(OH)(SO_3_)_2_]·H_2_O. J. Therm. Anal. Calorim. 2018, 132, 493–502. 10.1007/s10973-017-6901-4.

[ref3] Holló B. B.; Petruševski V. M.; Kovács G. B.; Franguelli F. P.; Farkas A.; Menyhárd A.; Lendvay G.; Sajó I. E.; Nagy-Bereczki L.; Pawar R. P.; Szilagyi I. M.; Bodis E.; Kótai L. Thermal and spectroscopic studies on a double-salt-type pyridine–silver perchlorate complex having κ1-O coordinated perchlorate ions. J. Therm. Anal. Calorim. 2019, 138, 1193–1205. 10.1007/s10973-019-08663-1.

[ref4] Kovacs G. B.; May N. V.; Bombicz P. A.; Klébert S.; Németh P.; Menyhárd A.; Novodárszki G.; Petrusevski V.; Franguelli F. P.; Magyari J.; Béres K.; Szilágyi I. M.; Kótai L. An unknown component of a selective and mild oxidant: structure and oxidative ability of a double salt-type complex having κ^1^O-coordinated permanganate anions and three- and four-fold coordinated silver cations. RSC Adv. 2019, 9, 28387–28398. 10.1039/C9RA03230D. PMC9071043 35529631

[ref5] Franguelli F. P.; Barta-Holló B.; Petrusevski V.; Sajó I. E.; Klébert S.; Farkas A.; Bódis E.; Szilágyi I. M.; Pawar R. P.; Kótai L. Thermal decomposition and spectral characterization of di[carbonatotetraamminecobalt(III)] sulfate trihydrate and the nature of its thermal decomposition products. J. Therm. Anal. Calorim. 2020, 10.1007/s10973-020-09991-3.

[ref6] Grant G. A.; Katz M. The oxidation of carbon monoxide by solid permanganate reagents. VII. Thermal decomposition of silver permanganate. Can. J. Chem. 1954, 32, 1068–1077. 10.1139/v54-142.

[ref7] Satava V.; Koerbl J. Analytische verwertung von silberpermanganat VII. Die thermische zersetzung des silberpermanganats. Collect. Czech. Chem. Commun. 1957, 22, 1380–1389. 10.1135/cccc19571380.

[ref8] Mahroua O.; Alili B.; Ammari A.; Bellal B.; Bradai D.; Trari M. On the physical and semiconducting properties of the crednerite AgMnO_2_ prepared by sol-gel auto- ignition. Ceram. Int. 2019, 45, 10511–10517. 10.1016/j.ceramint.2019.02.113.

[ref9] Koriche N.; Bouguelia A.; Mohammedi M.; Trari M. Synthesis and physical properties of new oxide AgMnO_2_. J. Mater. Sci. 2007, 42, 4778–4784. 10.1007/s10853-006-0741-0.

[ref10] Kótai L.; Sajó I.; Fodor J.; Szabó P.; Jakab E.; Argay G.; Holly S.; Gács I.; Banerji K. K. Reasons for and Consequences of the Mysterious Behaviour of Newly Prepared Hemipyridine Solvate of Bis(pyridine)silver(I) Permanganate, Agpy_2_MnO_4_*0.5py. Transition Met. Chem. 2005, 30, 939–943. 10.1007/s11243-005-6231-4.

[ref11] Kotai L.; Nemeth P.; Kocsis T.; Sajo I. E.; Pasinszki T.; Szilágyi M. A.; Kant R.; Pawar R. P.; Sharma P. K. A new route to synthesize controlled-size MMn_2_O_4_-type transition metal (M = Cd, Zn, Cu) nanomanganites. Nano Studies 2016, 13, 7–13.

[ref12] Kotai L.; Banerji K. K.; Sajo I.; Kristof J.; Sreedhar B.; Holly S.; Keresztury G.; Rockenbauer A. An unprecedented-type intramolecular redox reaction of solid tetraamminecopper(2+) bis(permanganate)-[Cu(NH_3_)_4_](MnO_4_)_2_) - A low-temperature synthesis of copper dimanganese tetraoxide-type (CuMn_2_O_4_) nanocrystalline catalyst precursors. Helv. Chim. Acta 2002, 85, 2316–2327. 10.1002/1522-2675(200208)85:8<2316::AID-HLCA2316>3.0.CO;2-A.

[ref13] Sajó I.; Kótai L.; Keresztury G.; Gács I.; Pokol G.; Kristóf J.; Soptrayanov B.; Petrusevski V.; Timpu D.; Sharma P. K. Studies on the Chemistry of Tetraamminezinc(II) Dipermanganate ([Zn(NH_3_)_4_](MnO_4_)_2_): Low-Temperature Synthesis of the Manganese Zinc Oxide (ZnMn_2_O_4_) Catalyst Precursor. Helv. Chim. Acta 2008, 91, 1646–1658. 10.1002/hlca.200890180.

[ref14] Kotai L.; Sajo I.; Jakab E.; Keresztury G.; Németh C.; Gács I.; Menyhárd A.; Kristóf J.; Hajba L.; Petrusevski V.; Ivanovski V.; Timpu D.; Sharma P. K. Studies on the chemistry of [Cd(NH_3_)_4_](MnO_4_)_2_. A low temperature synthesis route of the CdMn_2_O_4+x_ type NO_x_ and CH_3_SH sensor. Z. Anorg. Allg. Chem. 2012, 638, 177–186. 10.1002/zaac.201100467.

[ref15] Scagliari G.; Marangoni A. Isomorfismo fra perclorati e permanganati. Atti Real. Accad. Lincei, Rend. Class. Sci. fis. Ser. [5] 1914, 23, 12–14.

[ref16] Palatinus L.; Chapuis G. SUPERFLIP – a computer program for the solution of crystal structures by charge flipping in arbitrary dimensions. J. Appl. Crystallogr. 2007, 40, 786–790. 10.1107/S0021889807029238.

[ref17] Dolomanov O. V.; Bourhis L. J.; Gildea R. J.; Howard J. A. K.; Puschmann H. OLEX2: A Complete Structure Solution, Refinement and Analysis Program. J. Appl. Crystallogr. 2009, 42, 339–341. 10.1107/S0021889808042726.

[ref18] Sheldrick G. M. Crystal structure refinement with SHELXL. Acta Crystallogr., Sect. C: Struct. Chem. 2015, 71, 3–8. 10.1107/S2053229614024218. 25567568 PMC4294323

[ref19] Farrugia L. J. WinGXandORTEP for Windows: an update. J. Appl. Crystallogr. 2012, 45, 849–854. 10.1107/S0021889812029111.

[ref20] Bruni G.; Levi G. Gli ammoniacati dei sali d’argento. Gazz. Chim. Ital. 1916, 46, 17–42.

[ref21] Ephraim F. Ueber die Natur der Nebenvalenzen XIX. Ammoniakate des Silbers. Ber. Dtsch. Chem. Ges. 1918, 51, 706–710. 10.1002/cber.19180510185.

[ref22] Kótai L.; Gács L.; Sajó I. E.; Sharma P. K.; Banerji K. K. Beliefs and Facts in Permanganate Chemistry - An Overview on the Synthesis and the Reactivity of Simple and Complex Permanganates. Trends Inorg. Chem. 2011, 11, 25–104.

[ref23] Klobb T. Combinaisons de l’ammoniaque avec les permanganates metalliques. Compt. Rend. Hebd. Séanc. Acad. Sci. 1886, 103, 384–385.

[ref24] Nockemann P.; Meyer G. [Ag(NH_3_)_2_]ClO_4_: Kristallstrukturen, Phasenumwandlung, Schwingungsspektren. Z. Anorg. Allg. Chem. 2002, 628, 1636–1640. 10.1002/1521-3749(200207)628:7<1636::AID-ZAAC1636>3.0.CO;2-M.

[ref25] Mitscherlich E. Ueber die Mangansaeure, Uebermangansaeure, Ueberchlorsaeure und die Salze dieser Saeuren. Ann. Phys. 1832, 101, 287–302. 10.1002/andp.18321010608.

[ref26] Mitscherlich C. G. Ueber die Verbindungen des Quecksilbers. Ann. Phys. 1827, 85, 387–415. 10.1002/andp.18270850303.

[ref27] Kálmán A.; Párkányi L.; Argay G. Classification of the isostructurality of organic molecules in the crystalline state. Acta Crystallogr., Sect. B: Struct. Sci. 1993, 49, 1039–1049. 10.1107/S010876819300610X.

[ref28] Bereczki L.; Fogaca L.; Holló B. B.; Sajó I. E.; Bódis E.; Farkas A.; Menyhárd A.; Szilágyi I. M.; Kótai L. The existence and properties of the high-temperature polymorph of bis[diamminesilver(I)] sulfate: causes and effects. J. Coord. Chem., submitted.

[ref29] Geddes A. L.; Bottger G. L. The infrared spectra of silver ammine complexes. Inorg. Chem. 1969, 8, 802–807. 10.1021/ic50074a020.

[ref30] Svatos G. F.; Curran C.; Quagliano J. V. Infrared Absorption Spectra of Inorganic Coördination Complexes.V. The N-H Stretching Vibration in Coördination Compounds. J. Am. Chem. Soc. 1955, 77, 6159–6163. 10.1021/ja01628a019.

[ref31] Svatos G. F.; Sweeny D. M.; Mizushima S.-I.; Curran C.; Quagliano J. V. Infrared Absorption Spectra of Inorganic Coördination Complexes. XII. The Characteristic NH_3_ Deformation Vibrations of Solid Inorganic Complexes. J. Am. Chem. Soc. 1957, 79, 3313–3315. 10.1021/ja01570a004.

[ref32] Miles M. G.; Patterson J. H.; Hobbs C. W.; Hopper M. J.; Overend J.; Tobias R. S. Raman and infrared spectra of isosteric diammine and dimethyl complexes of heavy metals. Normal-coordinate analysis of (X_3_Y_2_)_2_Z ions and molecules. Inorg. Chem. 1968, 7, 1721–1729. 10.1021/ic50067a006.

[ref33] Kummer N.; Ragle J. L.; Weiden N.; Weiss A. Proton Magnetic Resonance Study of Molecular Motion in Solid Silver Sulfate Tetrammine, Ag_2_SO_4_ 4NH_3_. Z. Naturforsch., A: Phys. Sci. 1979, 34, 333–339. 10.1515/zna-1979-0309.

[ref34] Grinberg A. A.; Varshavskii Y. S. The frequency of coordinated ammonia deformation mode and its relationship with the chemical properties of transition metal ammonia complexes. Primenenie Molekulyarnoi spektroskopii v khimii; Nauka: Moscow, 1966; pp 104–107.

[ref35] Caulder S. M.; Stern K. H.; Carter F. L. J. Inorg. Nucl. Chem. 1974, 36, 234–235. 10.1016/0022-1902(74)80699-8.

[ref36] Chang F. M.; Jansen M. Ag_1.8_Mn_8_O_16_: Square Planar Coordinated Ag⊕ Ions in the Channels of a Novel Hollandite Variant. Angew. Chem., Int. Ed. Engl. 1984, 23, 906–907. 10.1002/anie.198409061.

[ref37] Chang F. M.; Jansen M. La première des hollandites d’argent. Rev. chim. Miner. 1986, 23, 48–54.

[ref38] Yoshida H.; Ahlert S.; Jansen M.; Okamoto Y.; Yamaura J.-I.; Hiroi Z. Unique Phase Transition on Spin-2 Triangular Lattice of Ag_2_MnO_2_. J. Phys. Soc. Jpn. 2008, 77, 74719–74719. 10.1143/JPSJ.77.074719.

[ref39] Schenck R.; Bathe A.; Keuth H.; Süss S. über die Aktivierung der Mefalle durch fremde Zusätze. IV Belträge zur Chemie des Silbers. Z. Anorg. Allgem. Chem. 1942, 249, 88–99. 10.1002/zaac.19422490108.

[ref40] Rienäcker G.; Werner K. Über ternaere oxyde des drei- und zweiwertigen Mangans mit ein un zweiwertigem Kupfer und Silber. Monatsber. Deut. Akad. Wiss. Berlin 1960, 499–505.

[ref41] Hein F.; Daniel W.; Schwedler H. Ueber die Umsetzung des Silberpermanganats mit Wasserstoff. Z. Anorg. Allgem. Chem. 1937, 233, 161–177. 10.1002/zaac.19372330207.

[ref42] Hein F. Zir Kentniss der Abbau- und Reduktionsprodukte des Silberpermanganates. Z. Anorg. Allgem. Chem. 1937, 235, 25 10.1002/zaac.19372350103.

[ref43] Katz M.; Riberdy R.; Grant G. A. The oxidation of carbon monoxide by solid silver permanganate reagents: VI. Reaction kinetics and adsorption of water vapor. Can. J. Chem. 1956, 34, 1719–1729. 10.1139/v56-224.

[ref44] Hein F.; Daniel W.; Bhr G. Ueber die Umsetzung von waessrigen AgMnO4-Lösungen mit Silber bzw. Silber-Gold-Legierungen. Z. Anorg. Allg. Chem. 1958, 296, 73–90. 10.1002/zaac.19582960110.

[ref45] Zobetz E. Die Kristallstruktur der isotypen Verbindungen KAg(NO_3_)_2_, NH_4_Ag(NO_3_)_2_ und RbAg(NO_3_)_2_. Monatsh. Chem. 1980, 111, 1253–1263. 10.1007/BF00903652.

[ref46] Béres K. A.; Petrusevski V.; Barta-Holló B.; Németh P.; Fogaca L.; Franguelli F. P.; Menyhard A.; Szilágyi I. M.; Kótai L. An enigmatic “decomposition intermediate” of [Ag(NH_3_)_2_]MnO_4_ - characterization of AgNO_3_·NH_4_NO_3_ double salt. Z. Anorg. Allgem. Chem. 2021, under submission

[ref47] De Bruijn T. J. W.; De Jong W. A.; Van den Berg P. J. Thermal decomposition of aqueous manganese nitrate solutions and anhydrous manganese nitrate. Part 1. Mechanism. Thermochim. Acta 1981, 45, 265–278. 10.1016/0040-6031(81)85087-3.

