# Supplementary material for: Solid-Phase Quasi-Intramolecular Redox Reaction of [Ag(NH3)2]MnO4: An Easy Way to Prepare Pure AgMnO2
Source: Inorg Chem. 2021 Mar 1;60(6):3749–60. doi: 10.1021/acs.inorgchem.0c03498 (PMC8034774; doi:10.1021/acs.inorgchem.0c03498)
Supplement: Supplementary file 1 — ic0c03498_si_001.pdf [file ic0c03498_si_001.pdf]

Supporting Information

A solid-phase quasi-intramolecular redox reaction of $[Ag(NH_3)_2]MnO_4$: an easy way to prepare pure $AgMnO_2$

Lara A. Fogaca[†‡], Éva Kováts[*], Gergely Németh[*], Katalin Kamarás[*], Kende A. Béres,[‡] Péter Németh,[‡,§] Vladimir Petruševski,["] Laura Bereczki,[⊥] Berta Barta Holló,[#] István E. Sajó,[@] Szilvia Klébert,[‡] Attila Farkas[%], Imre M. Szilágyi[†], and László Kótai[‡∇,]*

[†]Department of Inorganic and Analytical Chemistry, Budapest University of Technology and Economics, Műegyetem rakpart 3, Budapest, H-1111, Hungary
[‡]Institute of Materials and Environmental Chemistry, Research Centre for Natural Sciences, Magyar Tudósok krt 2, Budapest, 1117, Hungary
[*]Wigner Research Centre for Physics, Institute for Solid State Physics and Optics, Konkoly Thege u. 29-33, Budapest, H-1121, Hungary
[§]Department of Earth and Environmental Sciences, University of Pannonia, Egyetem út 10, Veszprém, 8200, Hungary
["]Faculty of Natural Sciences and Mathematics, Ss. Cyril and Methodius University, Skopje, North-Macedonia
[⊥]Chemical Crystallography Research Laboratory, Research Centre for Natural Sciences
[#]Department of Chemistry, Biochemistry and Environmental Protection, Faculty of Sciences, University of Novi Sad, Trg Dositeja Obradovića 3, Novi Sad, 21000, Serbia
[@]János Szentágothai Research Centre, University of Pécs, Ifjúság útja 20, Pécs, H-7624, Hungary
[%]Department of Organic Chemistry, Budapest University of Technology and Economics, Budapest, H-1111, Hungary
[∇]Deuton-X Ltd, Selmeci u2. 89, Érd, H-2030, Hungary

Corresponding author: László Kótai, kotai.laszlo@ttk.hu

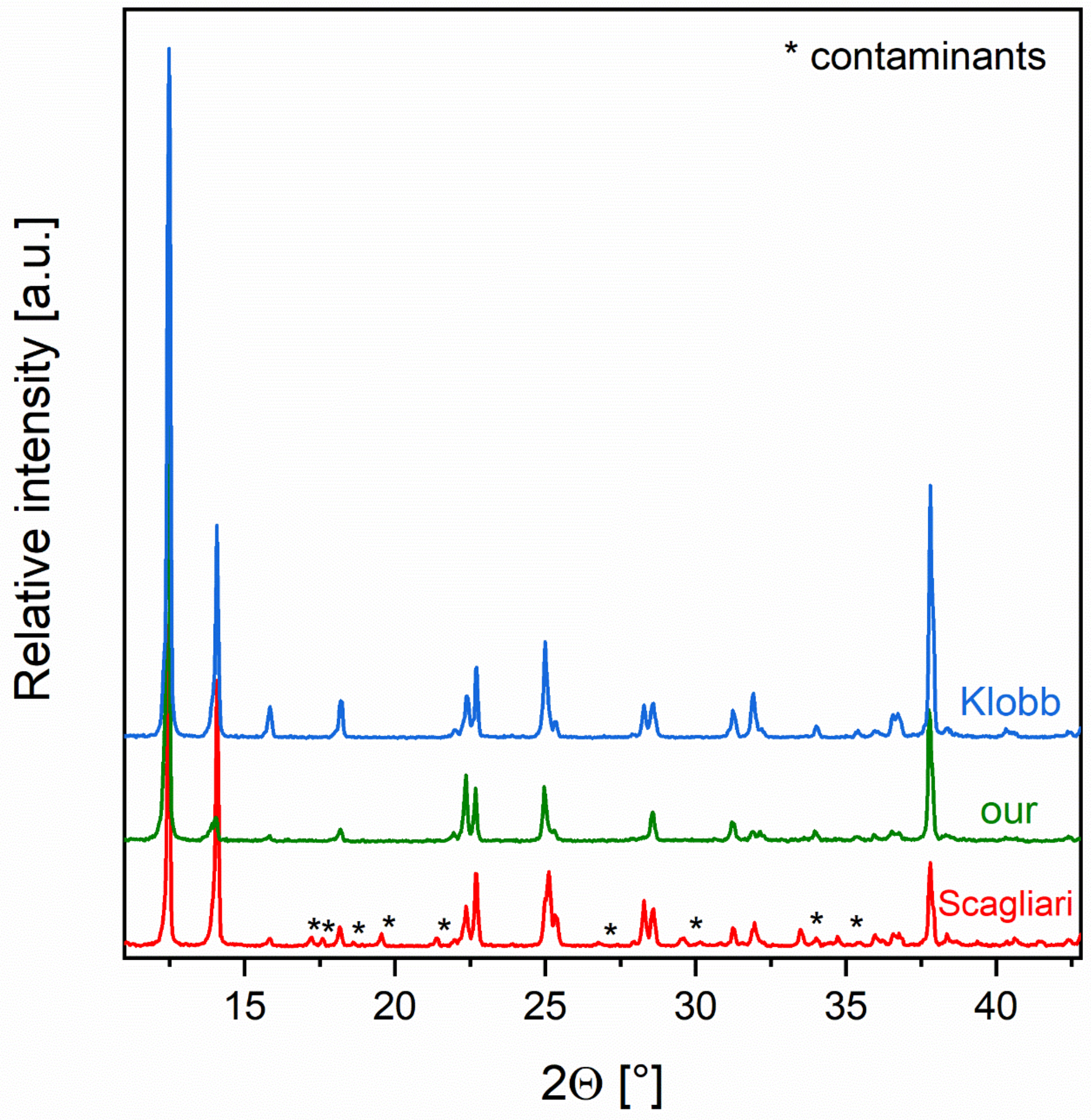

Figure S1. Powder X-ray diffractograms of products prepared by method of Klobb,[17] Scagliari and Marangoni [13] and in the reaction of $Ag(NH_3)_2NO_3$ and $NaMnO_4$.

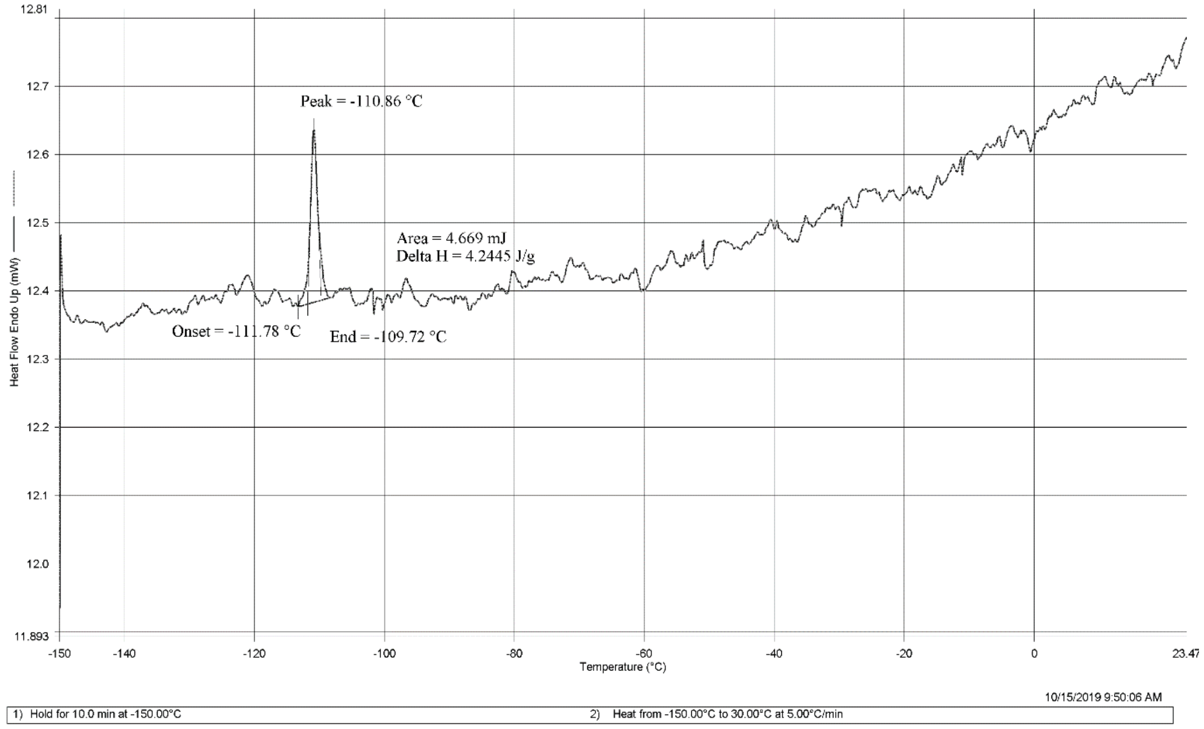

Figure S2. DSC Study on compound **1** between -150 and 25 °C

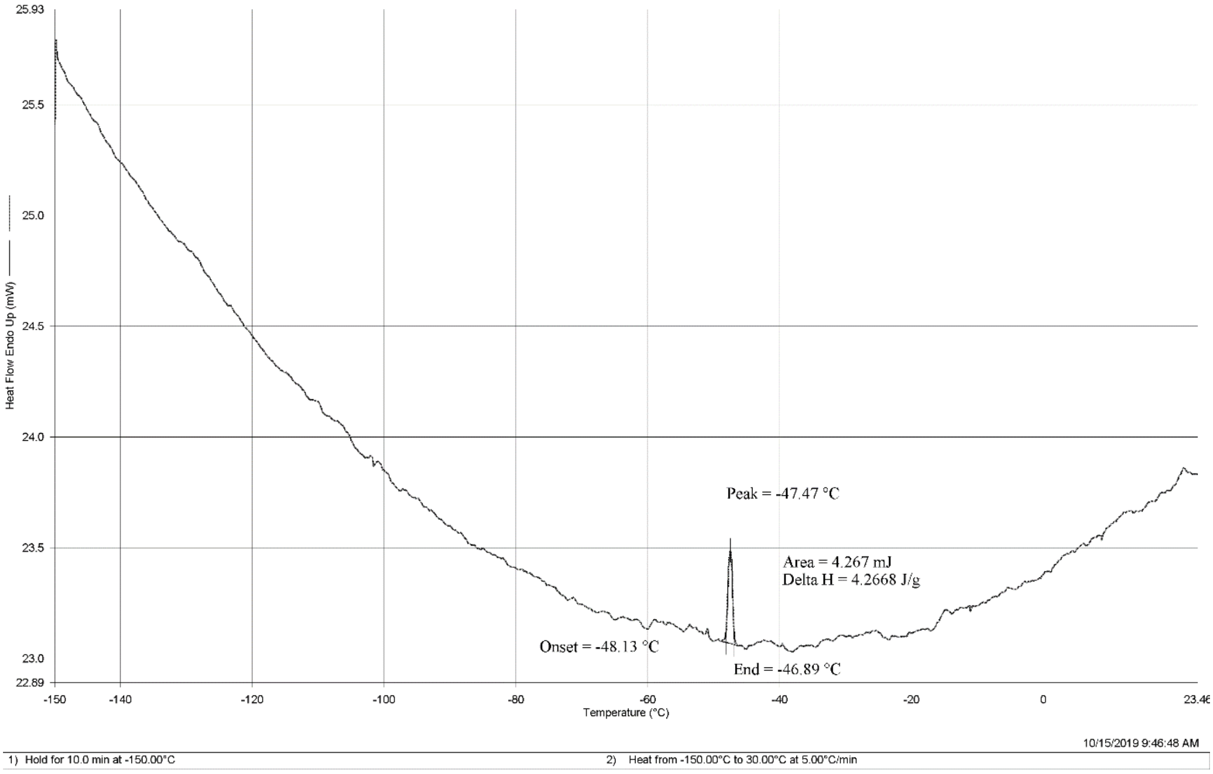

Figure S3. DSC Study on compound **1-ClO_4** between -150 and 25 °C

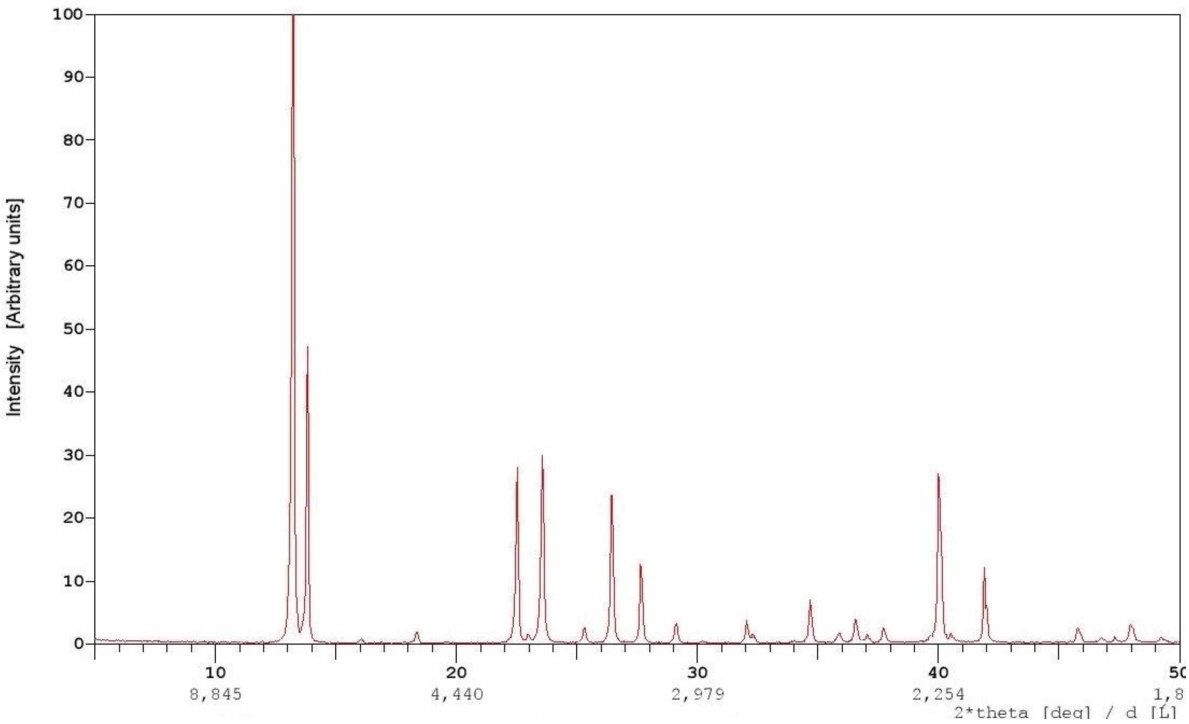

Figure S4. Powder XRD of orthorhombic HT-[Ag(NH_3)$_2$]ClO_4 (compound **1**-ClO_4).

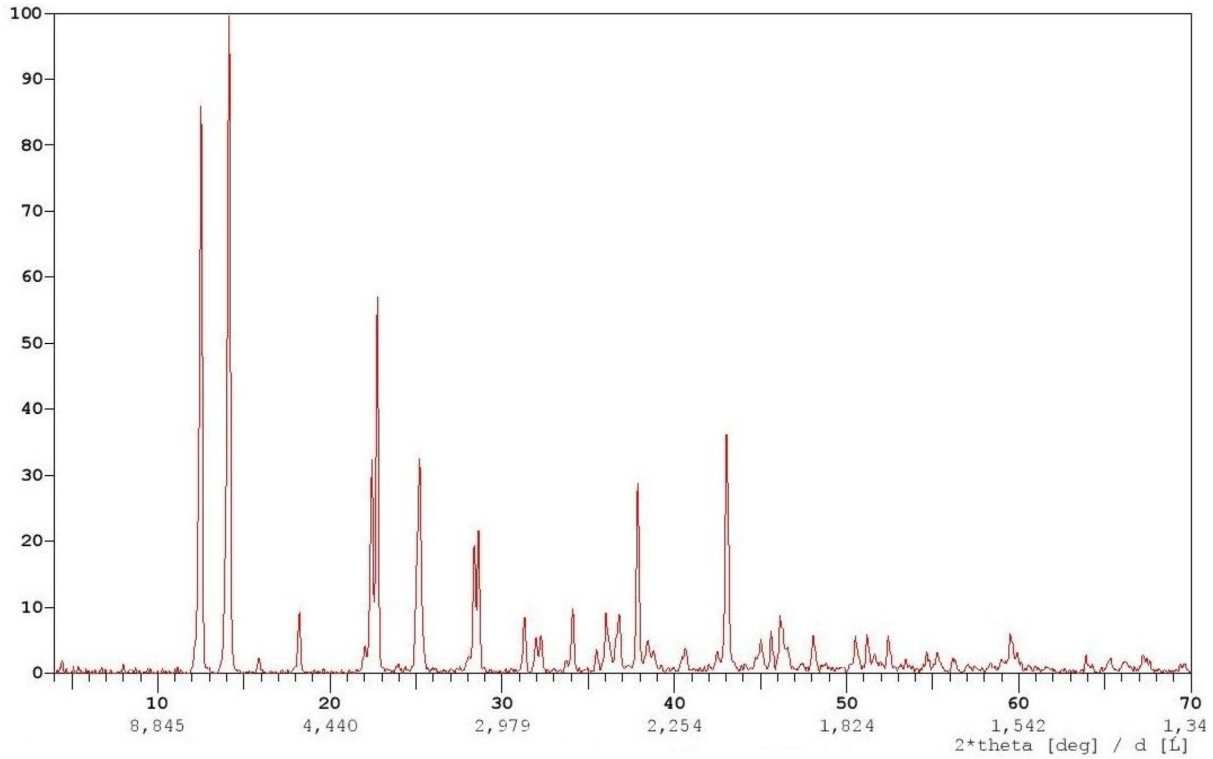

Figure S5. Powder XRD of monoclinic HT-$[Ag(NH_3)_2]MnO_4$ (compound **1**).

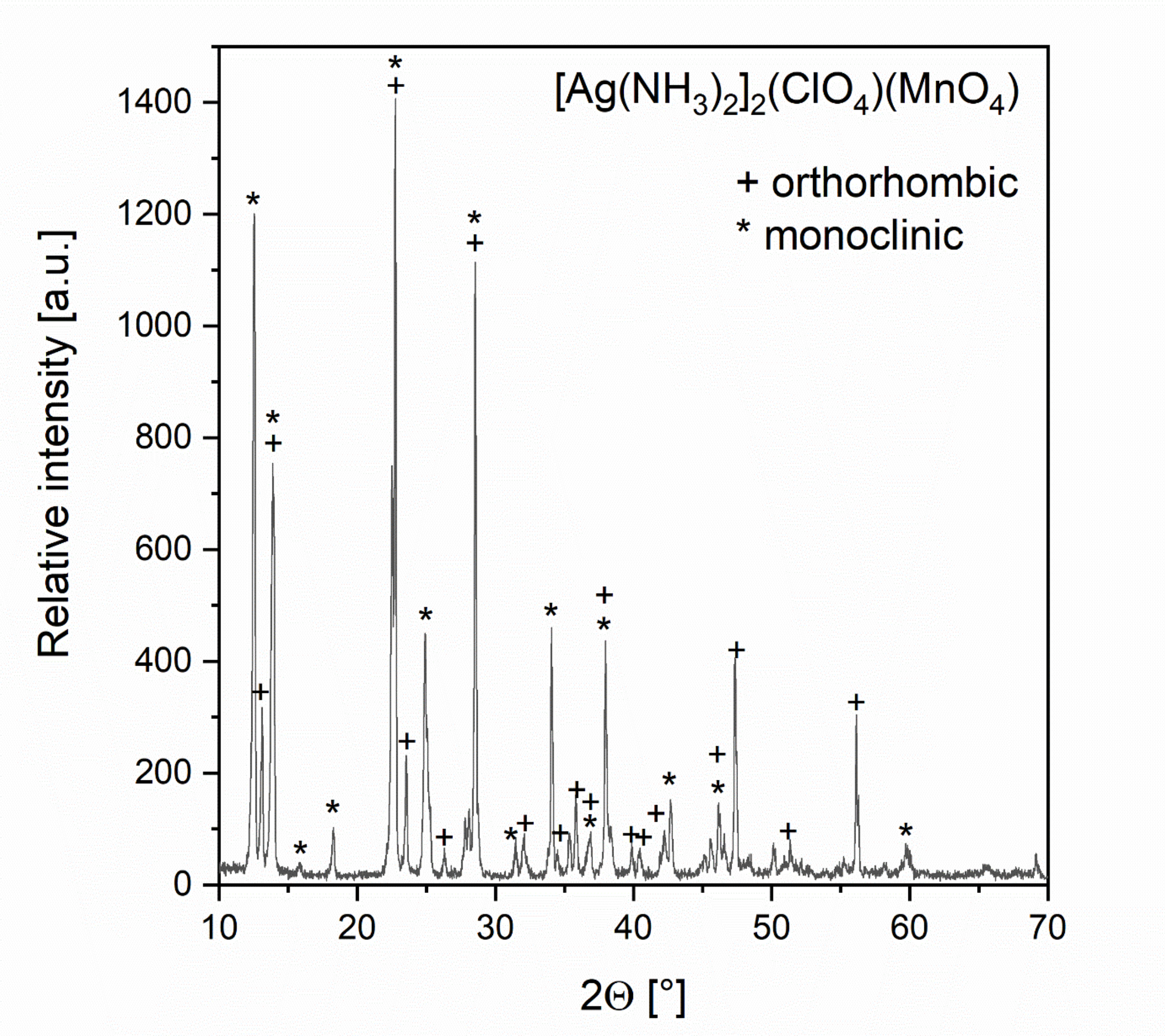

Figure S6. Powder XRD of solid solution contains ~ 28 and 72 mol % permanganate and perchlorate ions, respectively.

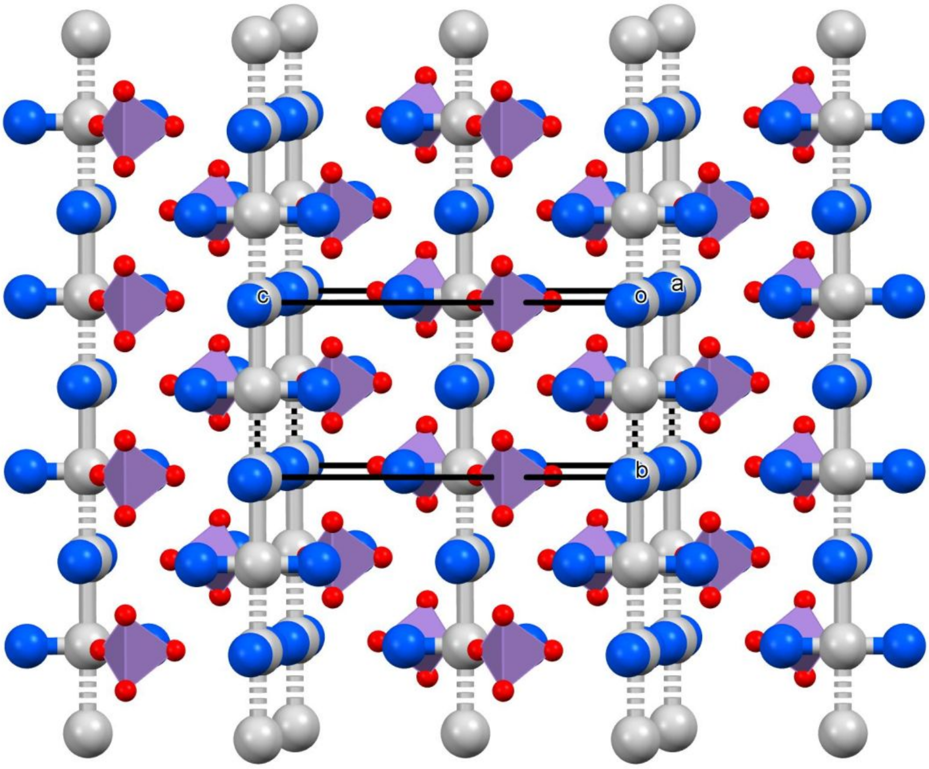

Figure S7. The packing of ions in the lattice of **HT-1** compound.

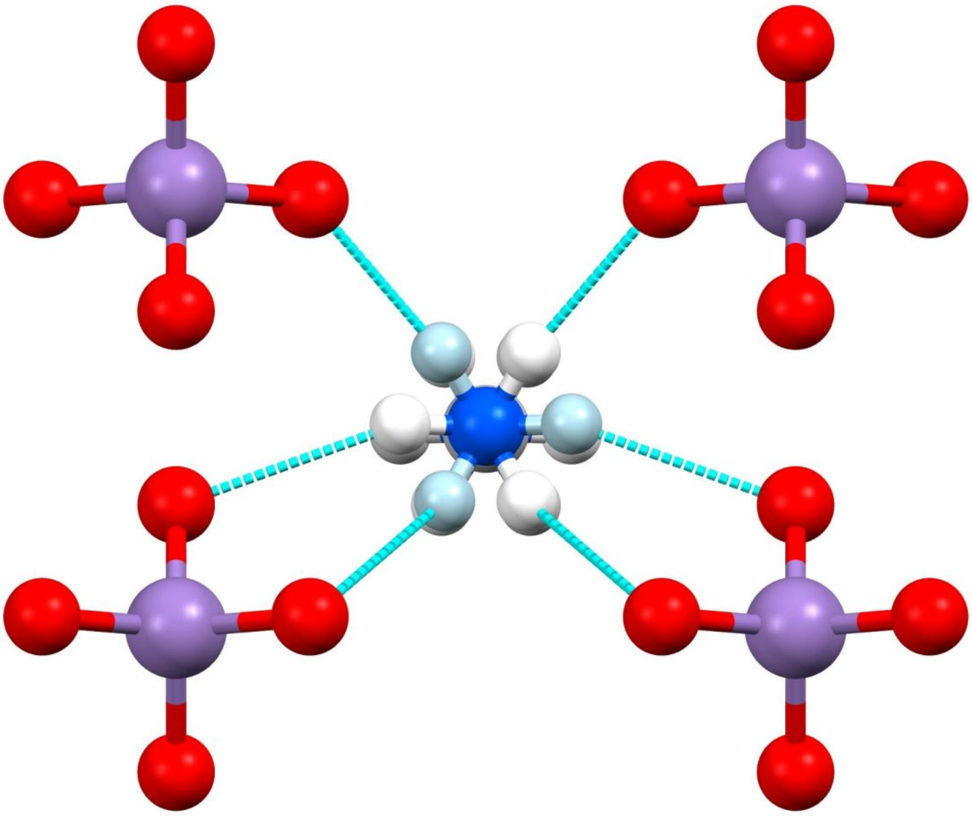

Figure S8. The disordered ammonia molecule and its hydrogen bond system in compounds **LT-1** and **HT-1**

a)

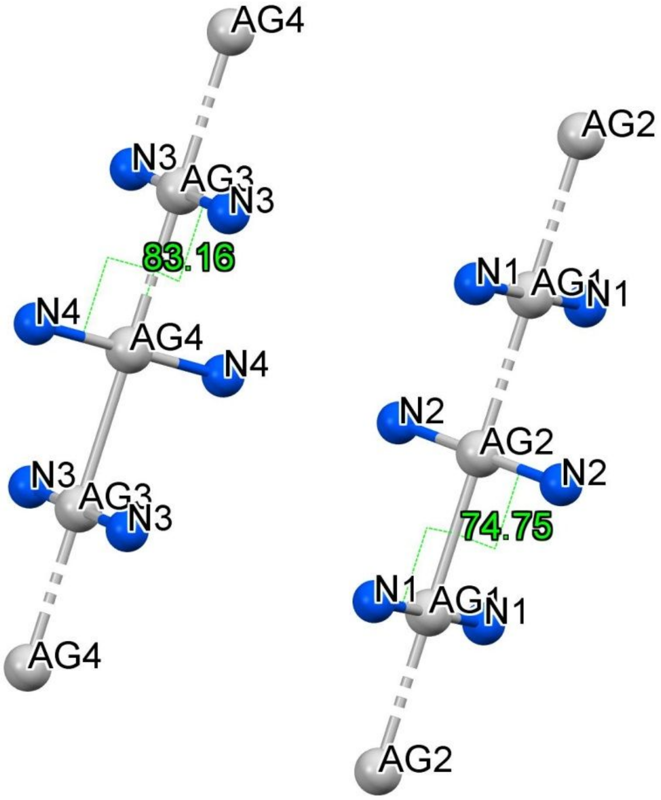

b)

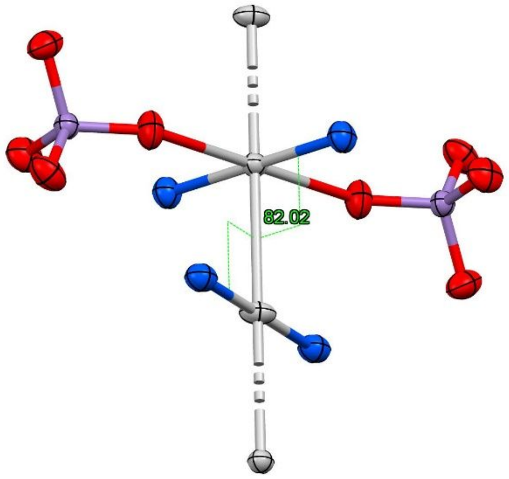

Figure S9. The torsion angles of diamminosilver(1+) units in compounds **LT-1**(a) and **HT1** (b)

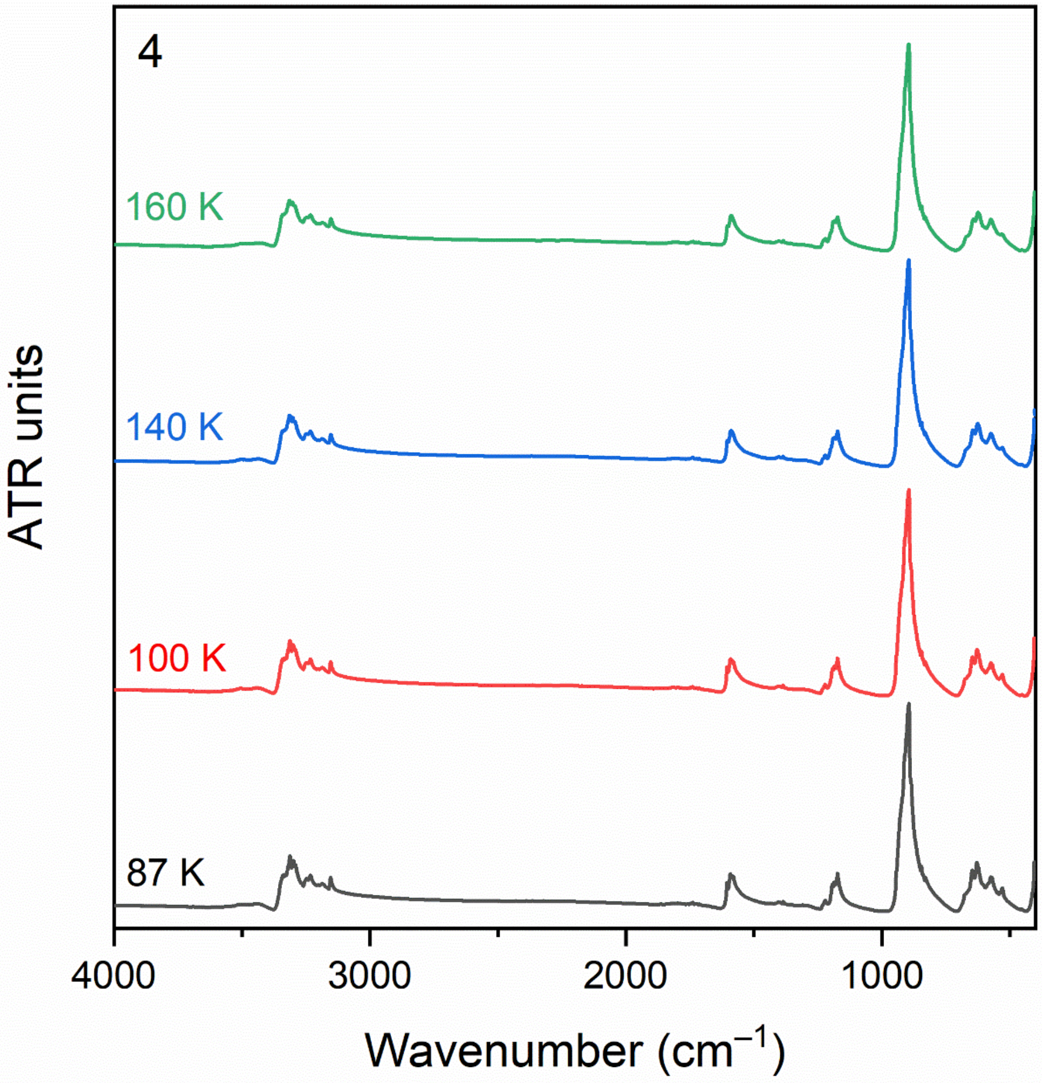

Figure S10. Temperature dependent IR spectra of compound **LT-1**

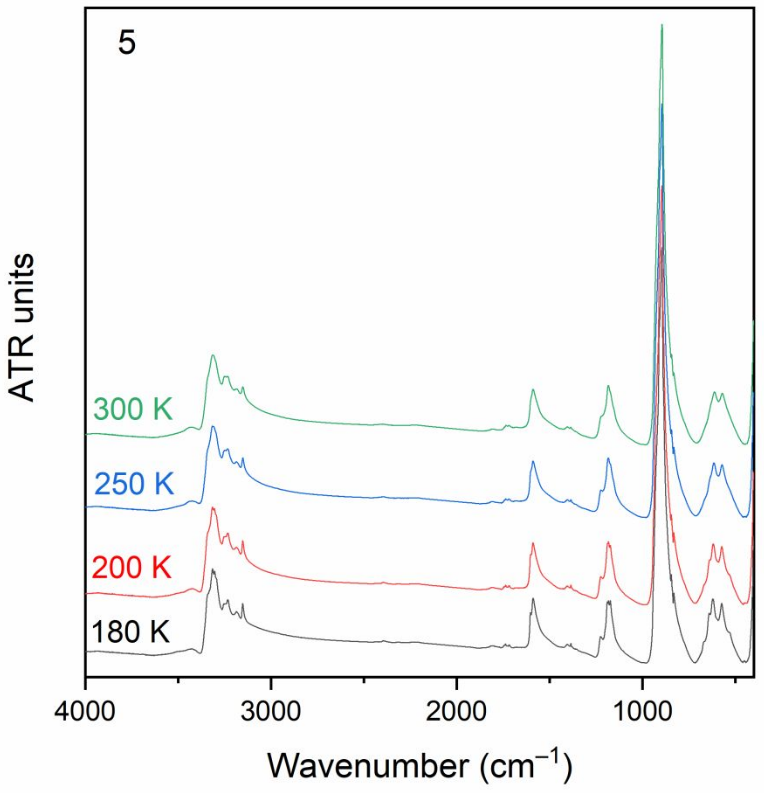

Figure S11. Temperature-dependent IR spectra of compound **HT-1**

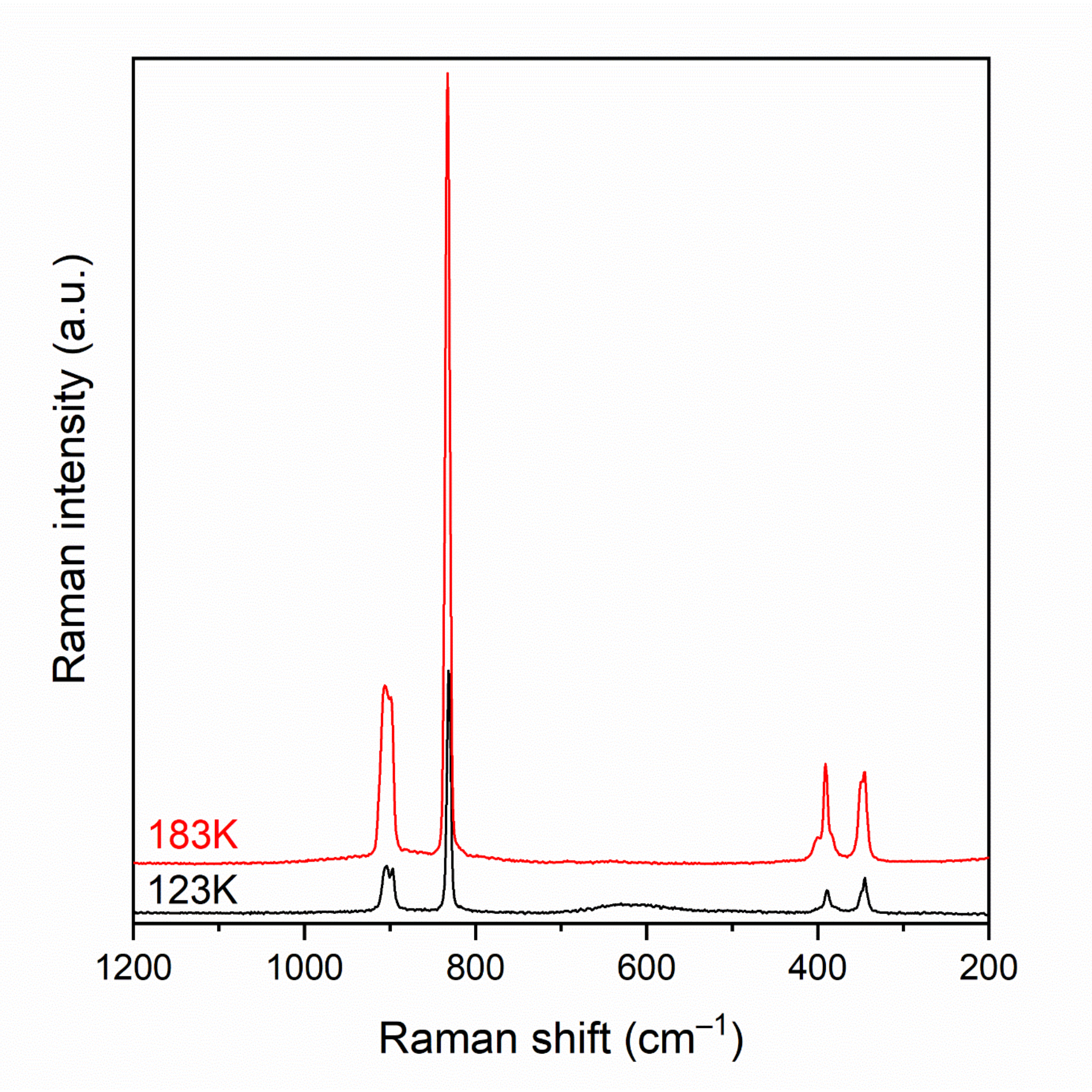

Figure S12. Raman spectra (532 nm excitation) of compounds **LT-1** and **HT-1** at 123 and 183 K, respectively.

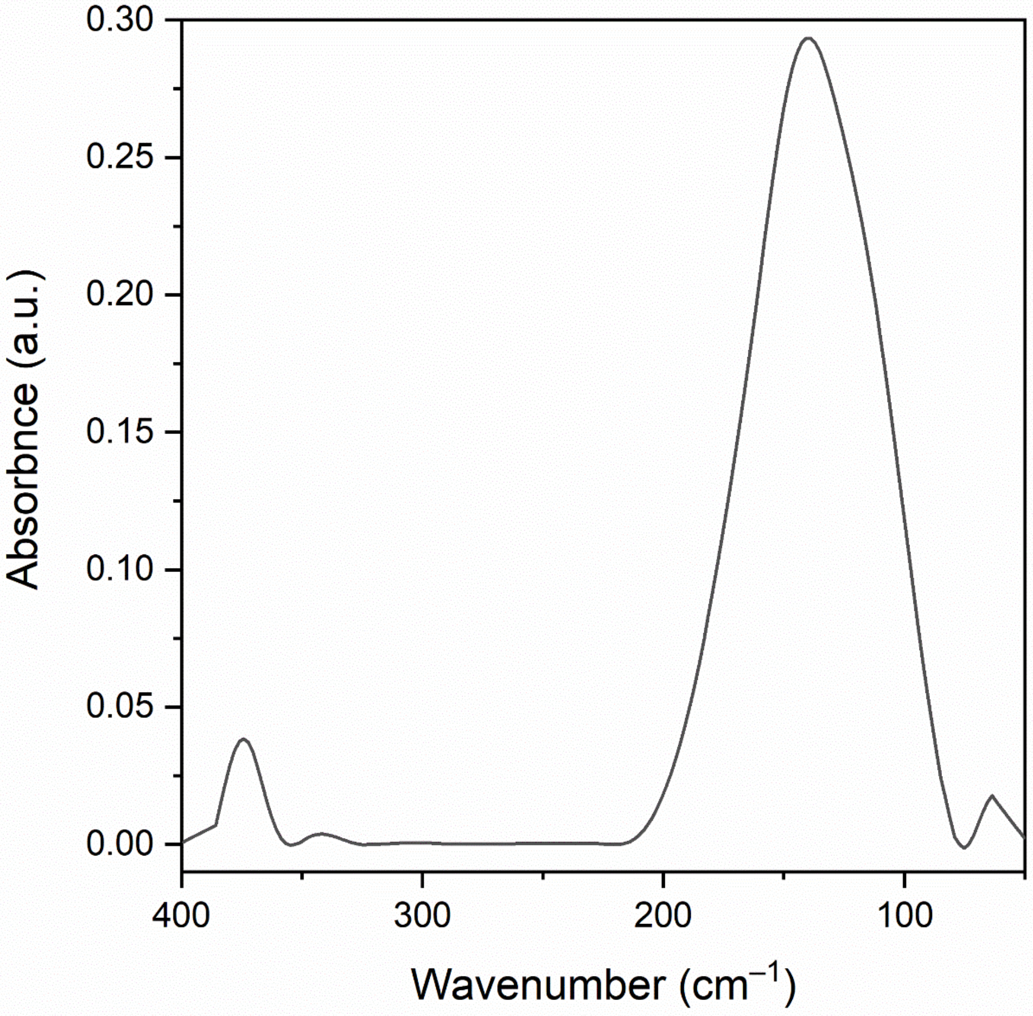

Figure S13. Far-IR spectrum of compound **HT-1**

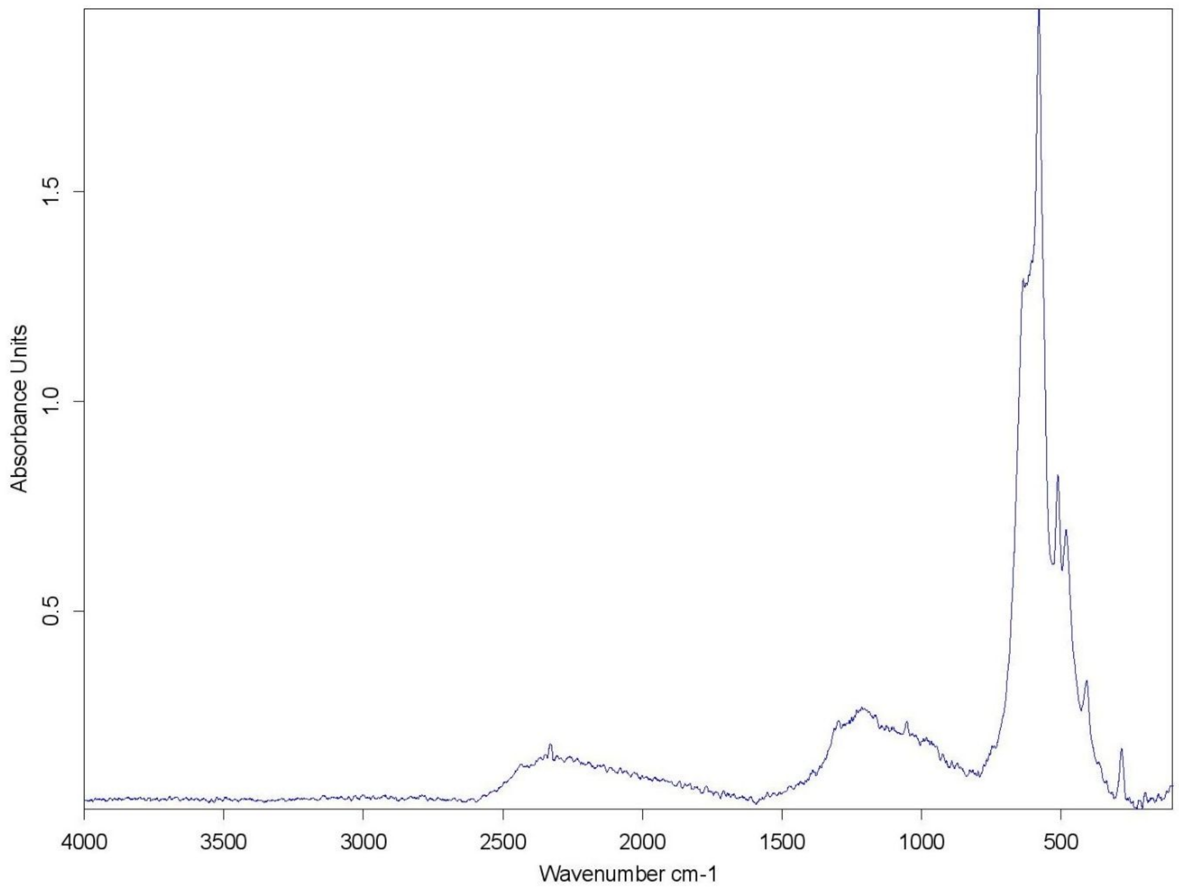

Figure S14. Raman spectrum of decomposition products formed from compound **HT-1** at 223 K during Raman measurement on irraditation with 532 nm laser light

Correlation analysis for polymorphs ***LT-1*** *and* ***HT-1***

The isolated permanganate ion has T_d symmetry, and accordingly, four Raman and two IR (F_2 species) active modes can be expected: ν_1 (symmetric stretching), ν_2 (symmetric bending), ν_3 (antisymmetric stretching) and ν_4 (antisymmetric bending). The symmetric bending mode is doubly (E), whereas the ν_3 and ν_4 modes are triply (F_2) degenerate under the molecular symmetry (T_d). In principle, after removing the degeneracy levels completely, 9 internal modes of permanganate ion can be expected.

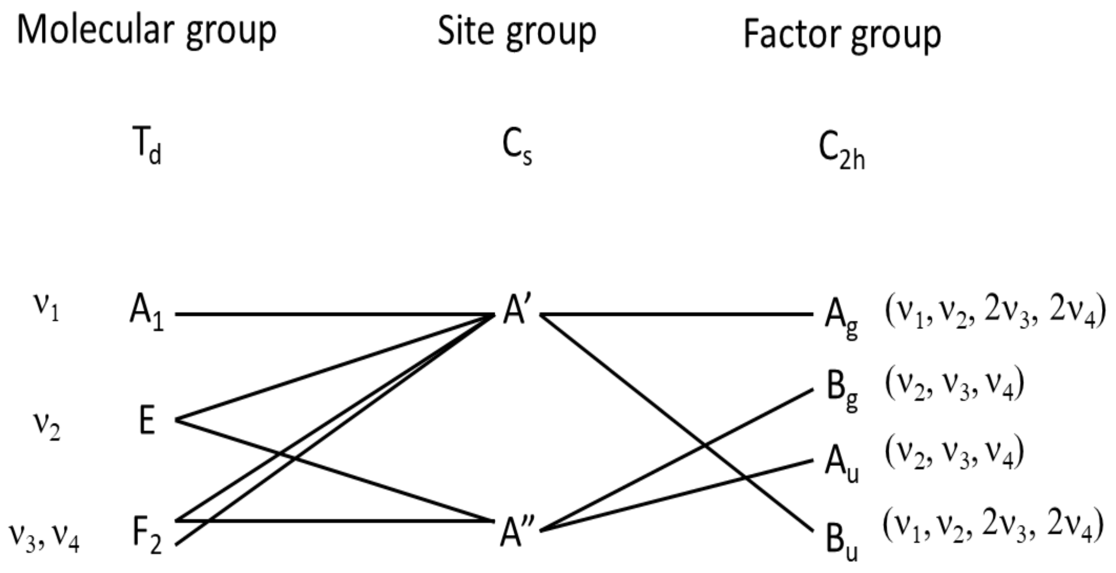

Figure S15. Correlation diagram for the **internal modes of permanganate ion in compounds LT-1** and **HT-1** (2 and 1 kind of permanganate ions, respectively)

The compound **LT-1** has two different permanganate ion types, thus the number of vibrations is double than that for compound **HT-1**. The hindered rotations and translations of the permanganate anions are both triply degenerate, under T_d symmetry. The total number of factor-group modes, due to the external MnO_4^- vibrations (hindered translations and hindered rotations), is equivalent with 2 x12=24 and 12 degrees of freedom for compounds **LT-1** and **HT-1**, respectively.

The complex cation ($Ag(NH_3)_2^+$) modes are decomposed into components of ammonia as ligand (C_{3v}) modes and to the translation of central silver ions . For the ammonia as a ligand, v_1 is the symmetric stretching, v_2 is the symmetric bending, v_3 is the antisymmetric stretching and v_4 is the antisymmetric bending modes. Both v_3 and v_4 are doubly degenerated (E) vibrations under C_{3v}. The total number of factor-group modes, due to the internal vibrations and 4 or 2 types of crystallographically different ammonia ligands, are 4 x 12 = 48 and 2 x 12 = 24 resulting in 48 and 24 vibrational degrees of freedom in compound **LT-1** and **HT-1**, respectively.

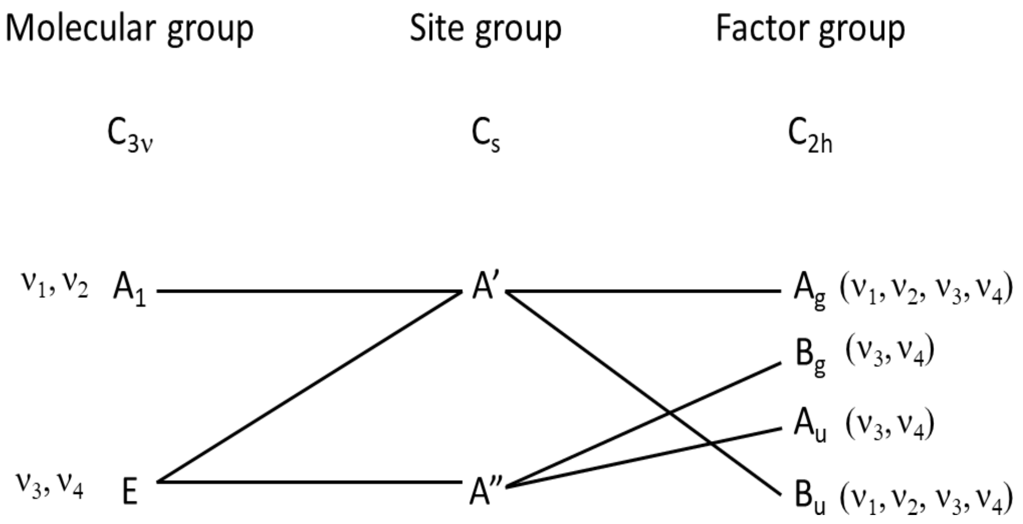

Figure S16. Correlation diagram for ammonia internal modes in compounds **LT-1** and **HT-1**

The external modes (T_{xy} and R_{xy}) are doubly degenerate modes under C_{3v}. The total number of factor-group modes due to the external vibrations is doubled and quadrupled (a consequence of two and four crystallographic types of NH_3) and are equivalent with 4 x 12 = 48 and 2 x 12 = 24 vibration degrees of freedom for compounds **LT-1** and **HT-1**, respectively.

Regarding the Ag^+-ions, there are 3 modes of acoustic origin out of the total of 48 (compound **LT-1**) and 24 (compound **HT-1**) external modes, which belong to species $A_u + 2B_u$. 45 and 21 optical modes of translational origin, 72 and 36 optical modes of rotational origin and 84 and 42 optical modes due to internal vibrations for compounds **LT-1** and **HT-1**, respectively.

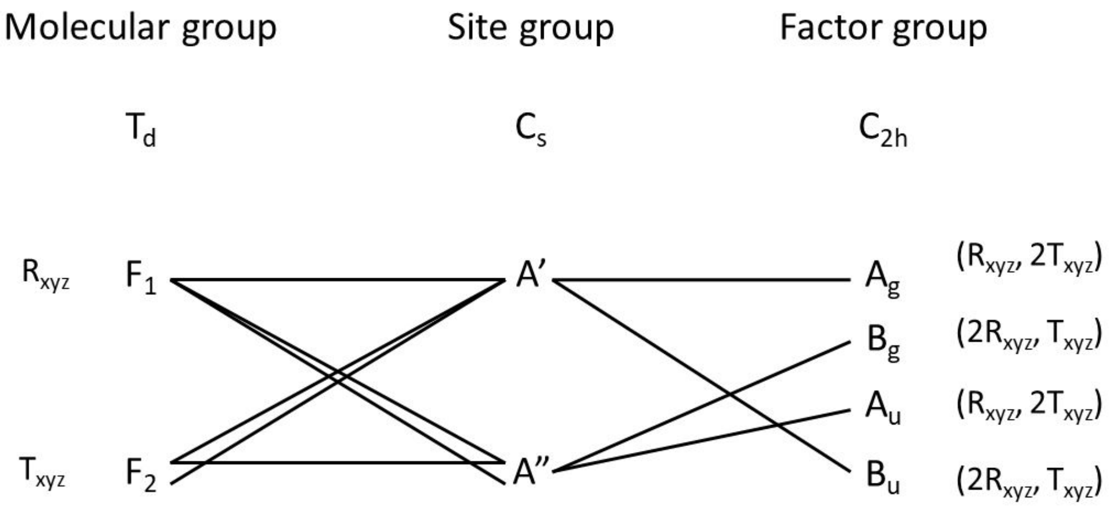

Figure S17. Correlation diagram for permanganate ion external modes in compounds **LT-1** and **HT-1** (2 and 1 kind of permanganate ions, respectively)

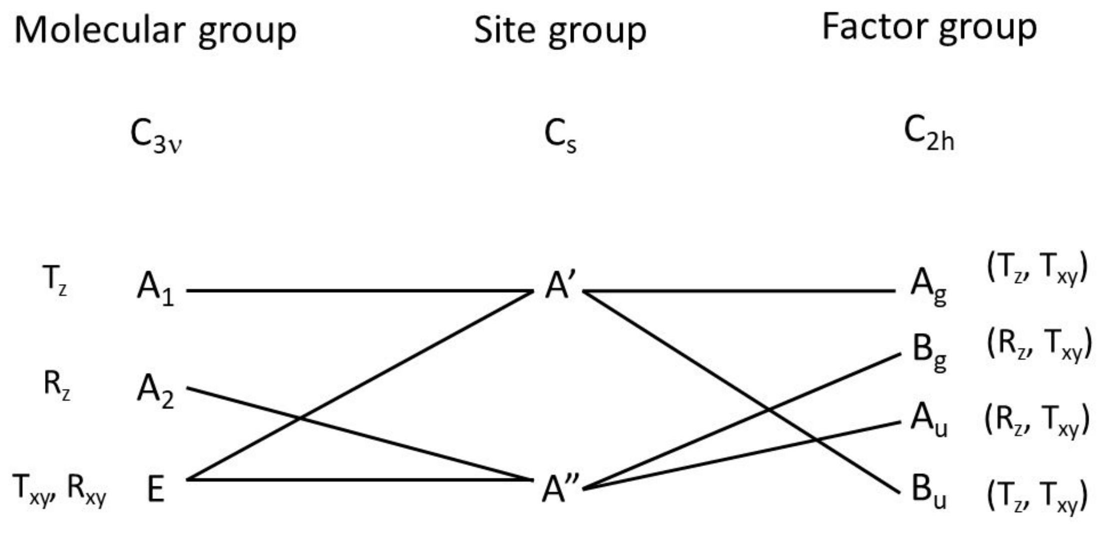

Figure S18. Correlation diagram for ammonia external modes in compounds **LT-1** and **HT-1** (4 and 2 kind of ammonia, respectively)

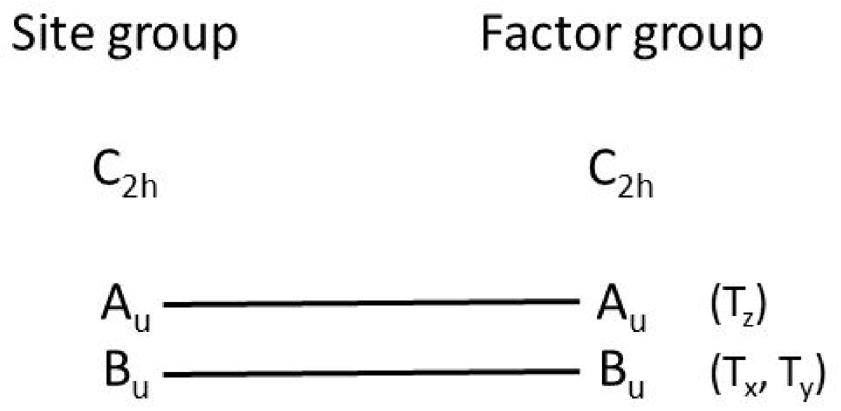

Figure S19. Correlation diagram for silver(1+) external modes in compounds **LT-1** and **HT-1**

UV spectroscopic results

The diffuse UV reflectance spectra of the solid solution of compound **HT-1** (1 %) in **HT-1-ClO$_4$** had a very broad band system, which might contain the orbital allowed **($^1A_1$-$^1T_2$)**(t_1-2e) (480-580 nm) and ($^1A_1$-$^1T_2$)(t_1-t_2) (320-380 nm).[29] The permanganate ($^1A_1$-$^1T_2$)(3t_2-2e) transition is expected to be a weak band between 380 and 320 nm.[30] The site symmetry of permanganate ion in compound **HT-1-ClO$_4$** is C_s, thus $^1T_2$ level splits into three states of symmetry, A", A'(1) and A'(2), and this splitting can further complicate the band system. In the UV range below 260 cm^{-1} the Ag 4d-5s transition and the N_p-Ag_{5s} or O_p-Ag_{5s} LMCT transitions could give signal.

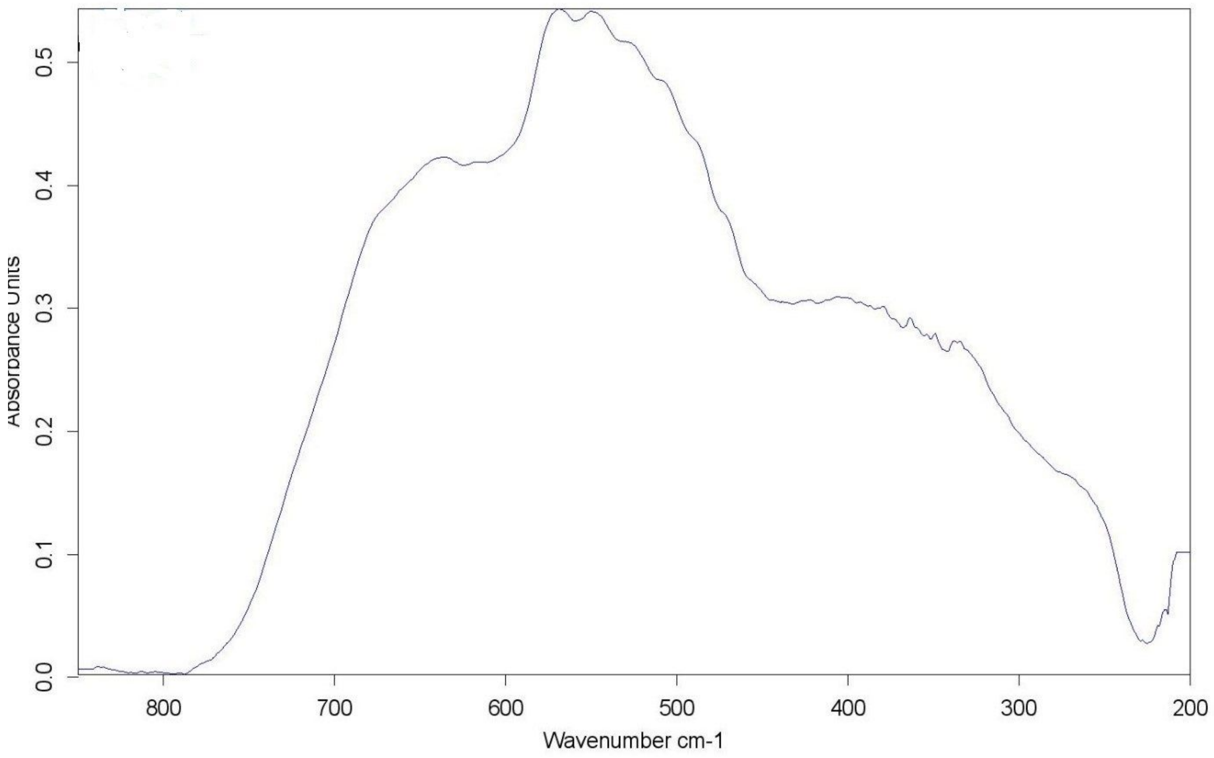

Figure S20. Diffuse reflectance UV spectrum of compound **HT-1**

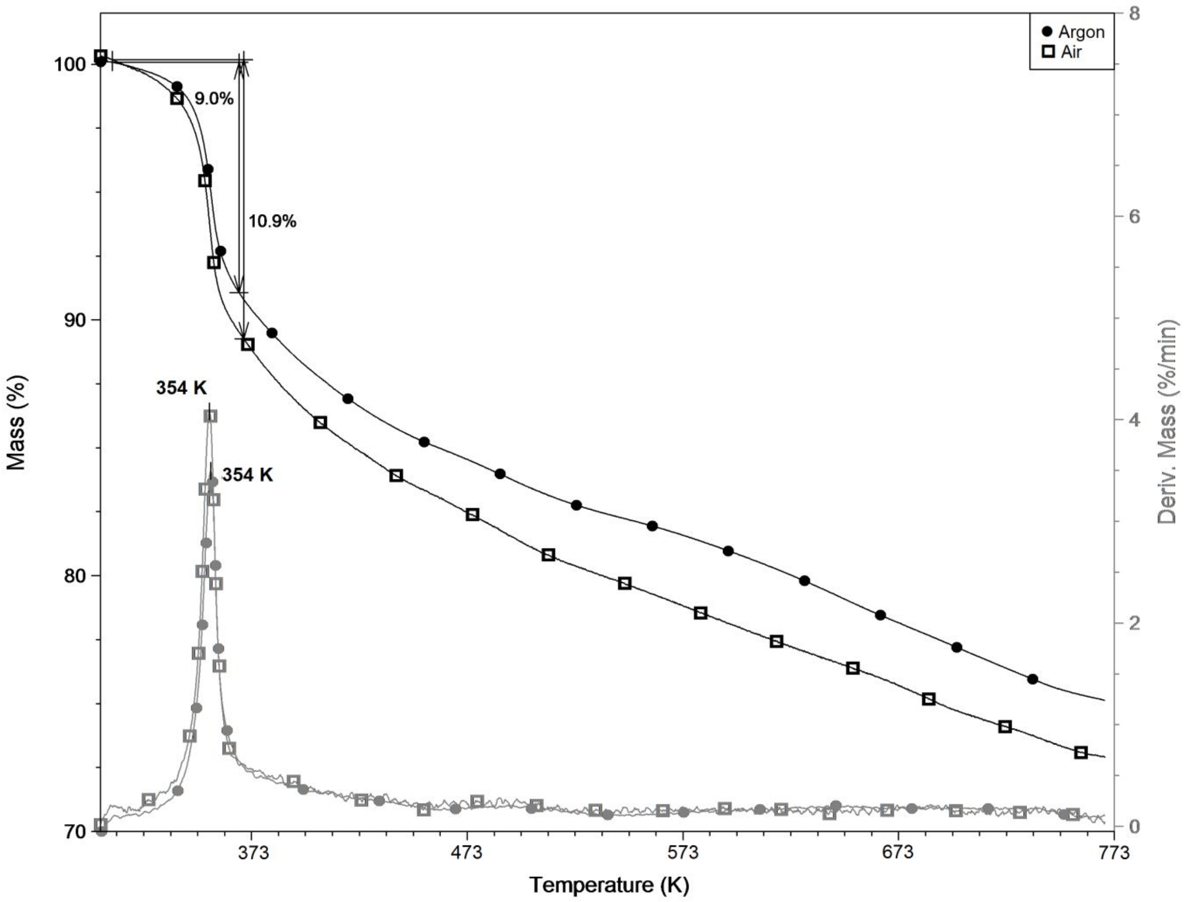

Figure S21. Thermal decomposition curves of compound **HT-1** in argon and air atmosphere.

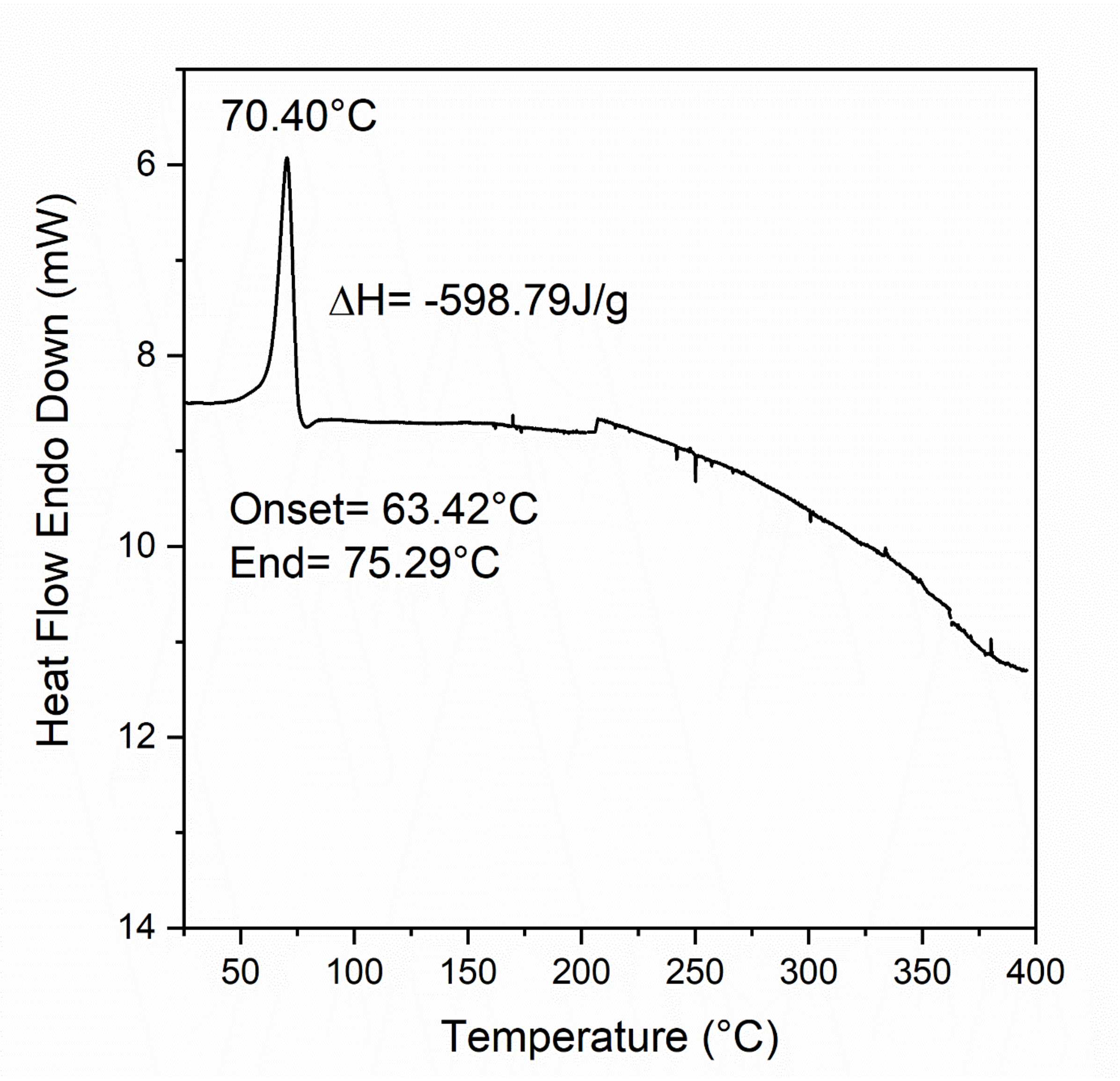

Figure S22. DSC of compound **HT-1** in N_2 atmosphere

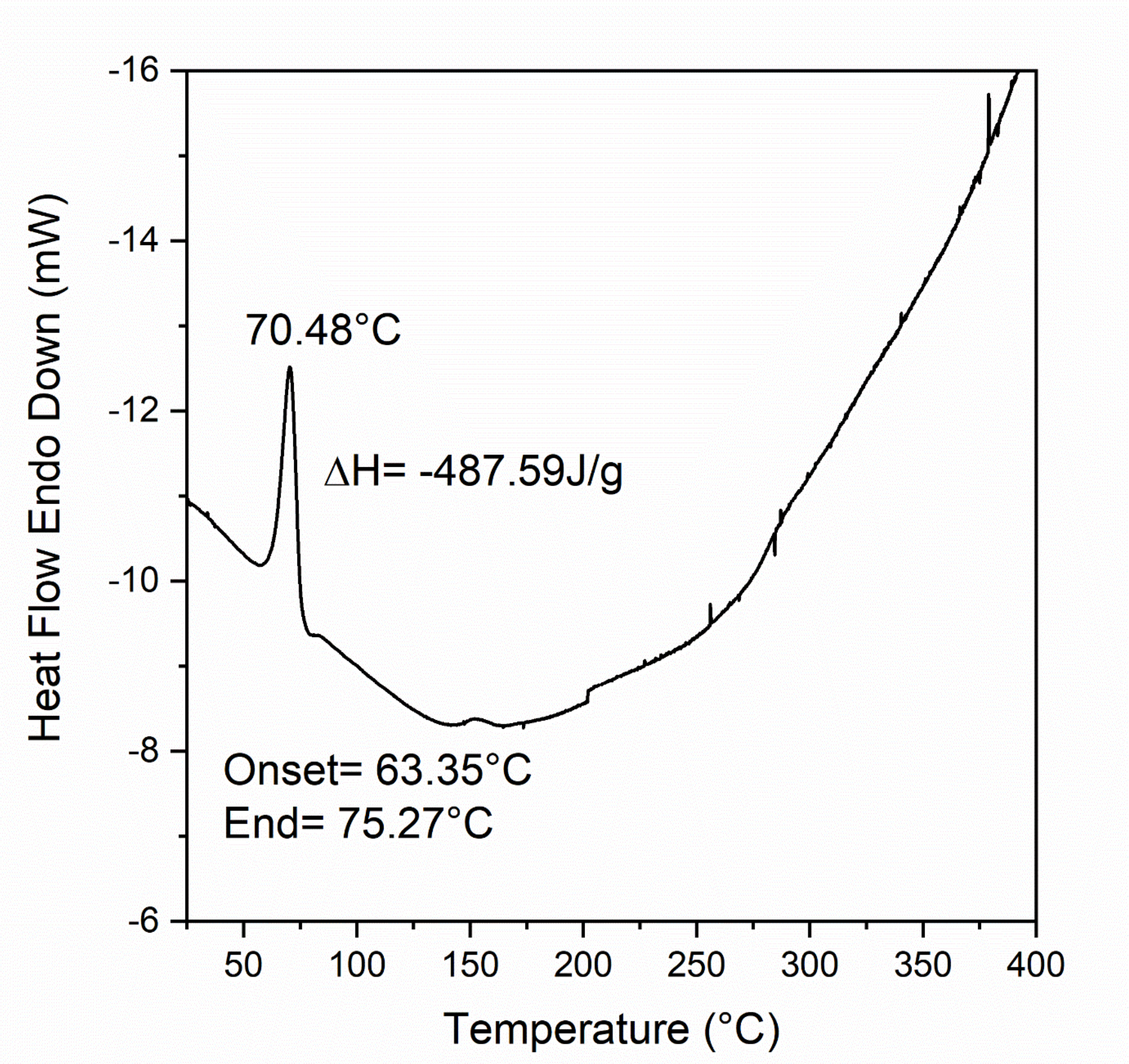

Figure S23. DSC of compound **HT-1** in O2 atmosphere

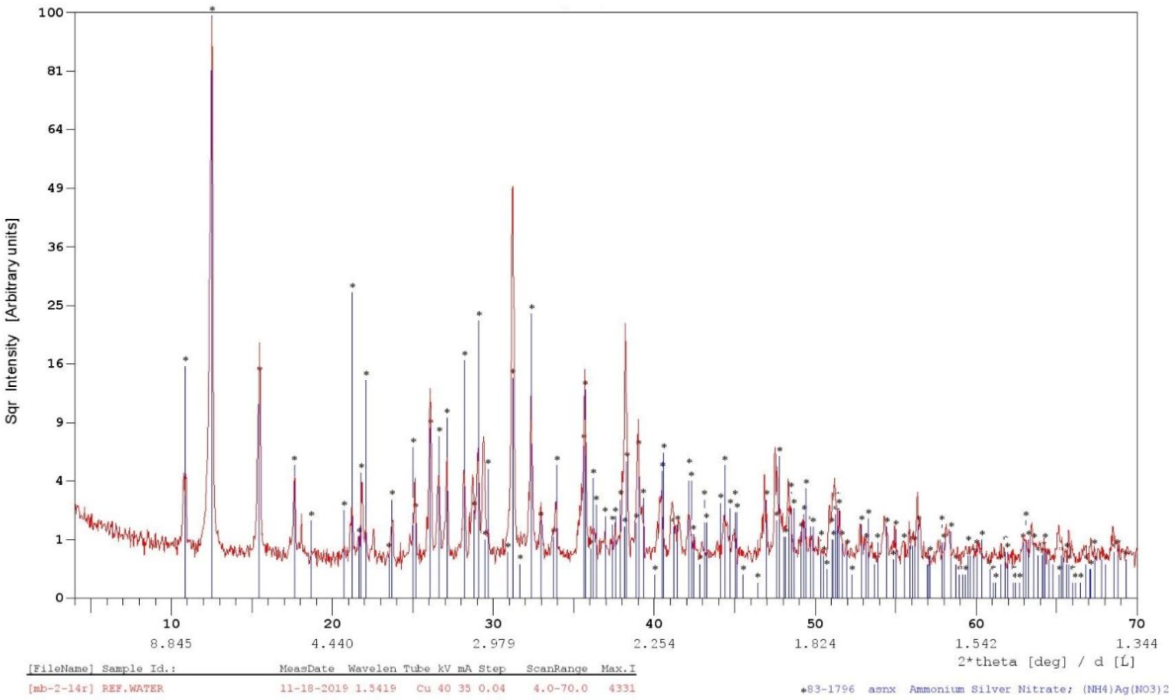

Figure S24. XRD of the evaporation **product obtained from the aq. extract formed in controlled temperature (under benzene) decomposition of** compound **HT-1**.

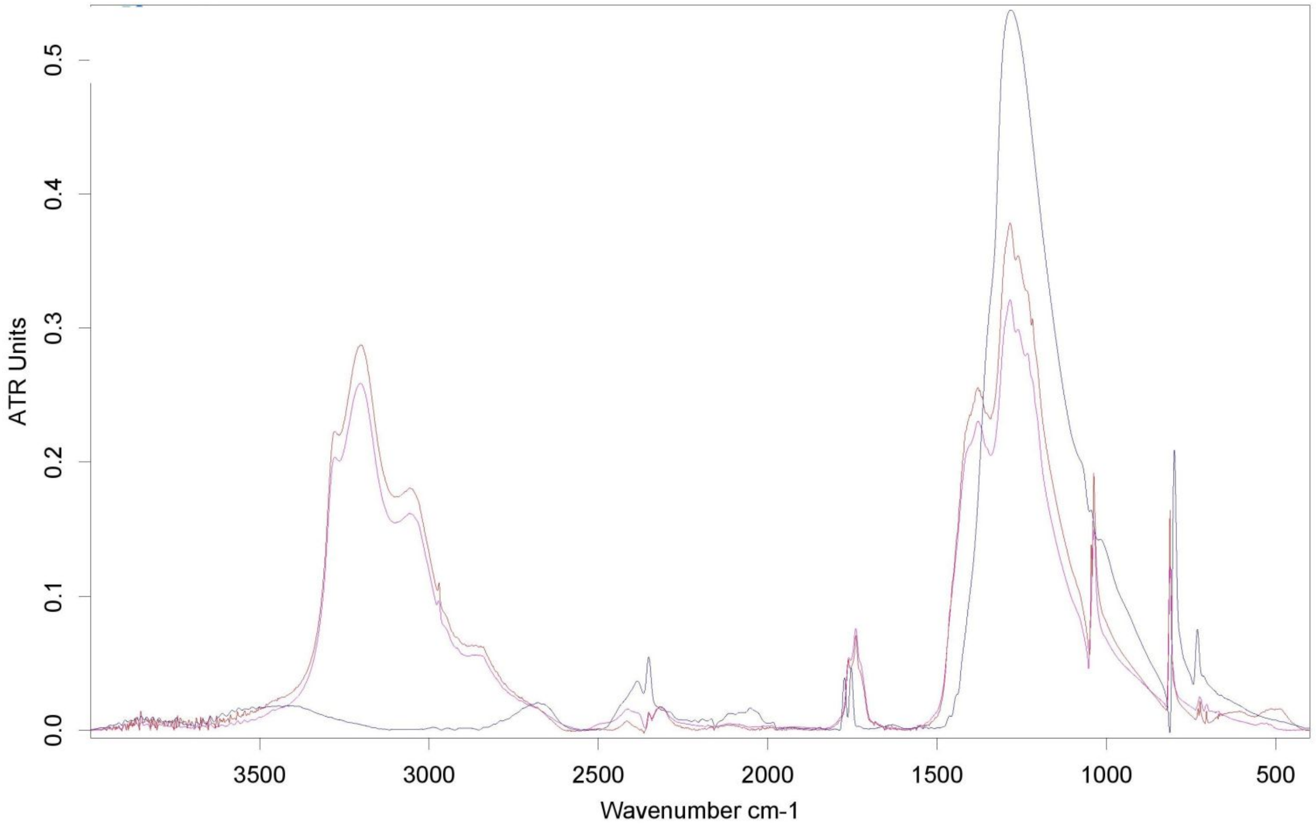

Figure S25. IR spectra of as-synthesized NH4NO3. AgNO3 (purple), AgNO3 (blue), and the decomposition product of compound **HT-1** at **125 °C (red**).

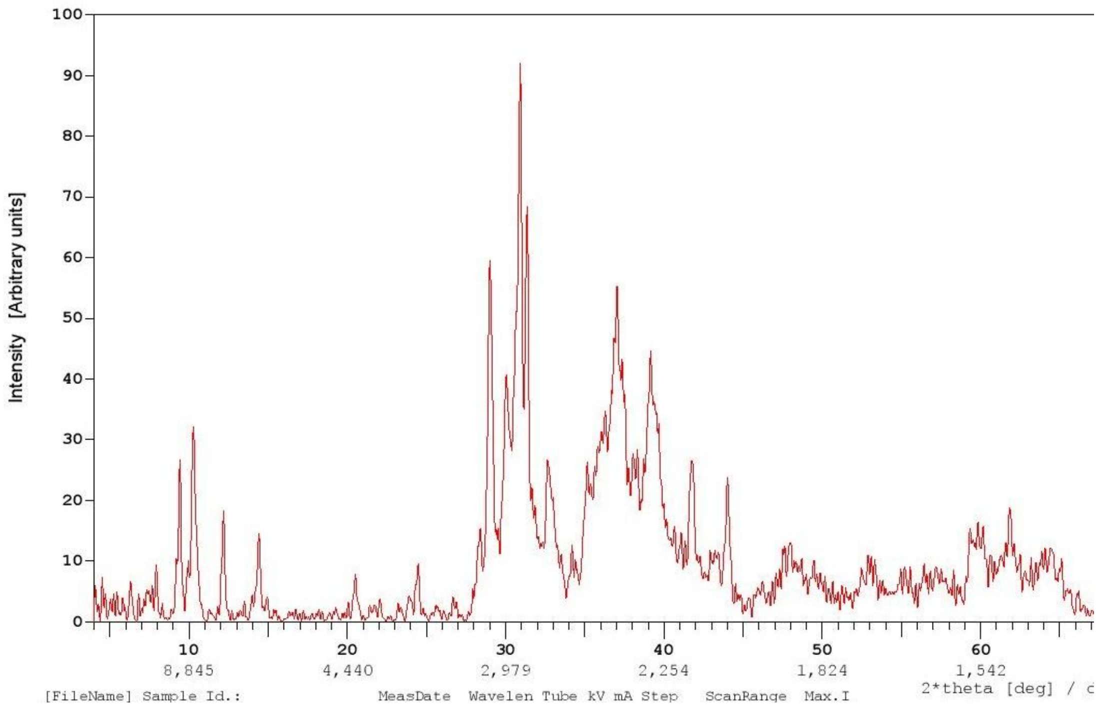

Figure S26. XRD of the decomposition product of compound **1 HT** after heating at **700 °C.**

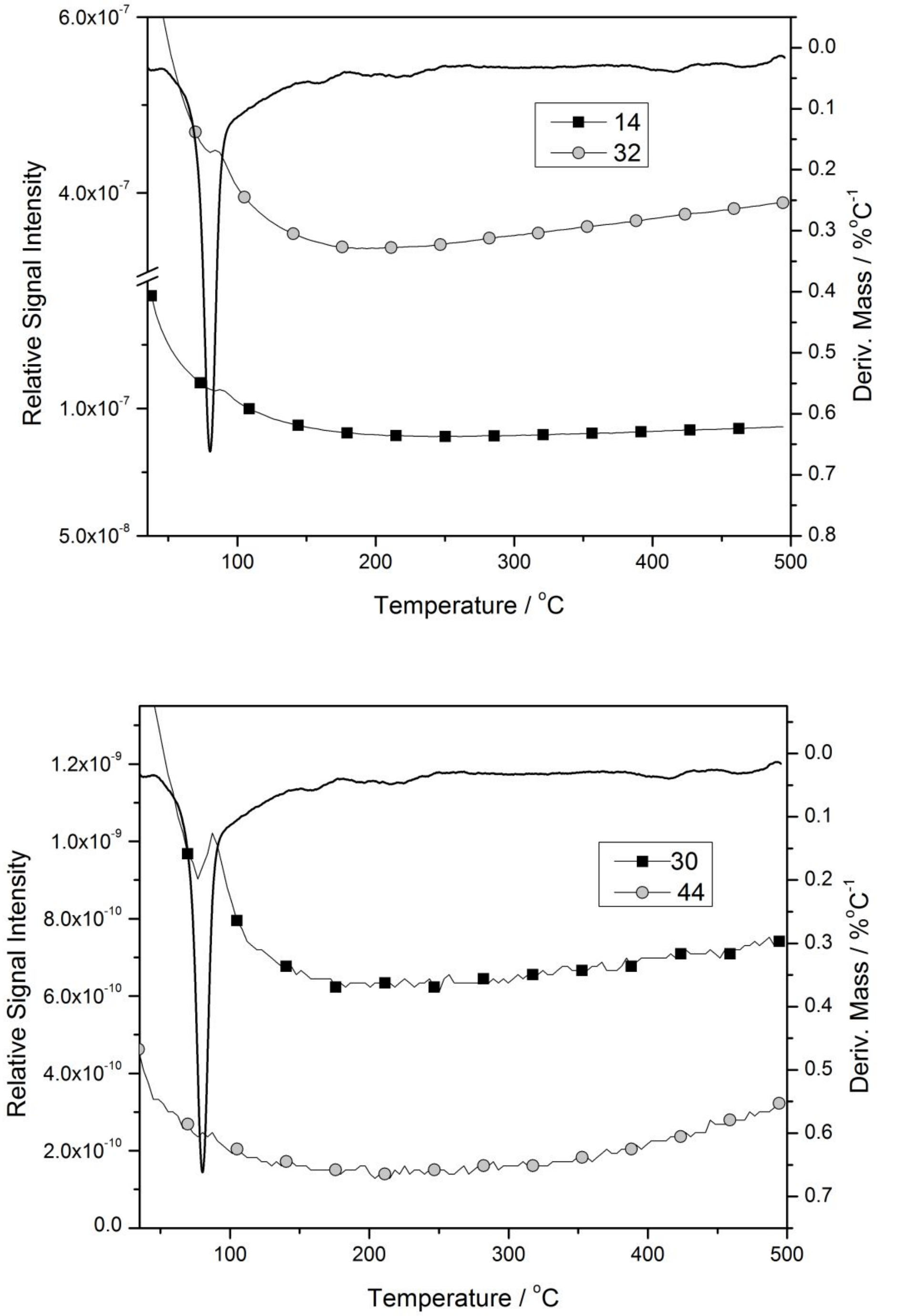

Figure S27. TG MS of compound 1 under air: 14 (N^+) and 32 (O_2^+), 30 (NO^+) and 44 (N_2O^+).

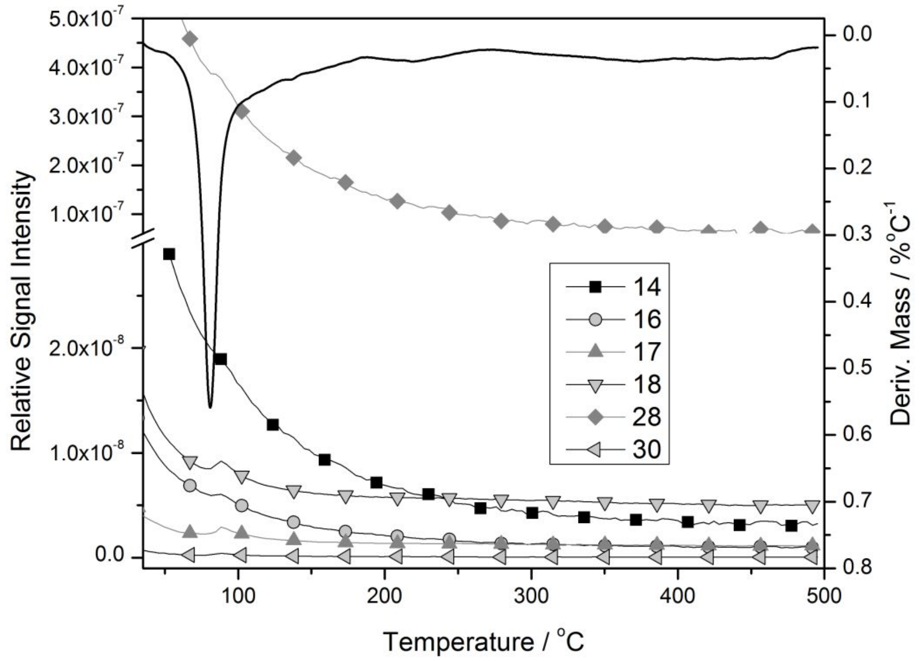

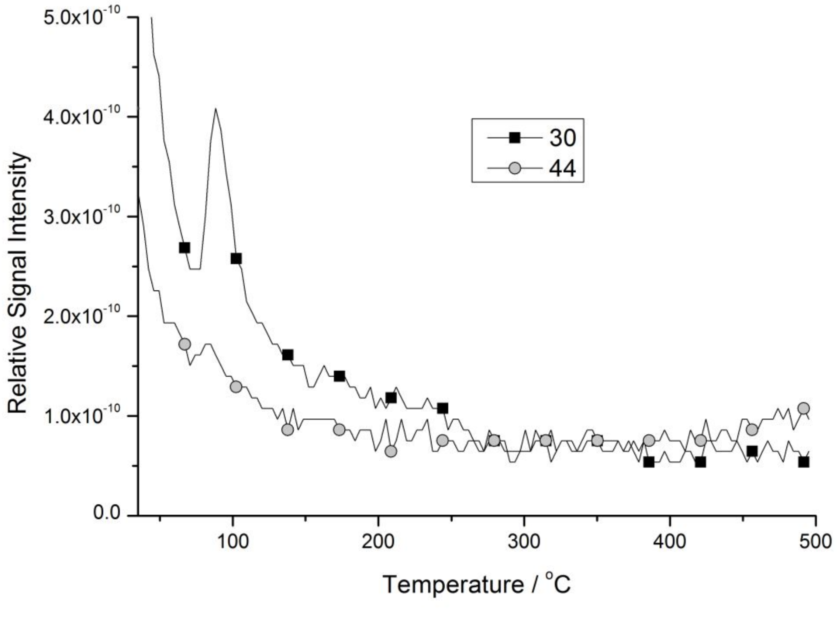

Figure S28. TG MS of compound **1** under argon: 14 (N^+), 15 (NH^+), 16 (NH_2^+, O^+), 17 (NH_3^+, OH^+), 18 (H_2O^+), 28 (N_2^+), 30 (NO^+) and 44 (N_2O^+).

Polymorphic crystal structures of diammine silver(I) permanganate measured at 100 K and 180 K.

Single crystal structures of two polymorphic modifications of $[Ag(NH_3)_2]MnO_4$ complex were determined at 100 K (**LT-1**) and 180 K (**HT-1**) respectively using MoK_α radiation. Both modifications crystallize in the monoclinic crystal system. The low temperature modification (**LT-1**) has the lower *P*2/m symmetry, which is a maximal non-isomorphic symmetry subgroup of the space group of the high temperature modification (**HT-1**) which is *I*2/m. **LT-1** is isomorphous to the known structure of $[Ag(NH_3)_2]ClO_4$ complex. The asymmetric unit of **LT-1** contains four quarter silver(I) cations, four half ammonia ligands and two half permanganate anions. The contents of the asymmetric unit of **HT-1** are one half of that of **LT-1** due to its higher symmetry. The unit cells of the two modifications are quite similar (ESI Table 1 and ESI Figure 29).

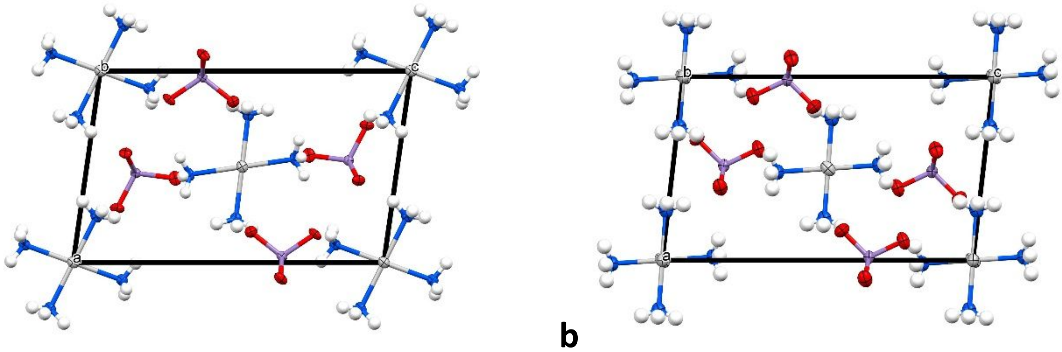

a **b**

Figure S29.. Comparison of the crystal packing of **M-1** (a) and **M-2** (b) (view from the direction of the *b* crystallographic axis)

The silver ions form infinite chains parallel to the *b* crystallographic axis in both structures. The Ag-Ag distance is 3.010 Å for **LT-1** and 3.034 Å for **HT-1**. The silver cations have two coordinated ammonia molecules and the ammonia hydrogens are disordered over two positions in both structures with 1:1 occupancy. Two permanganate anions are coordinated to the silver in both structures giving rise to an octahedral coordination around the silver ion (Figure S30) and to a three-dimensional coordination network. The atomic planes of nitrogen and oxygen atoms are perpendicular to the silver chain in all cases. the N-Ag-O angles are listed in ESI Table 2. In the **HT-1** structure, the Ag-N bonds are lying on the *a* and *c* unit cell axes while in **LT-1**, the Ag-N bonds are tilted from the unit cell axis directions.

The Ag-Ag chains coincide with the two-fold rotation axes, the Ag-N bonds are lying on mirror planes and the Ag ions sit on inversion centres in both structures. All of the permanganate anions are cut in half by mirror planes. In the high temperature modification parallel to the 2-fold rotation axes, 2-fold screw axes appear, which connects the Ag-Ag chains to each other. Besides, between every two mirror planes, a glide plane appears which

connects the Ag coordination sphere to each other. Thus, the permanganate anions are related by inversion centres.

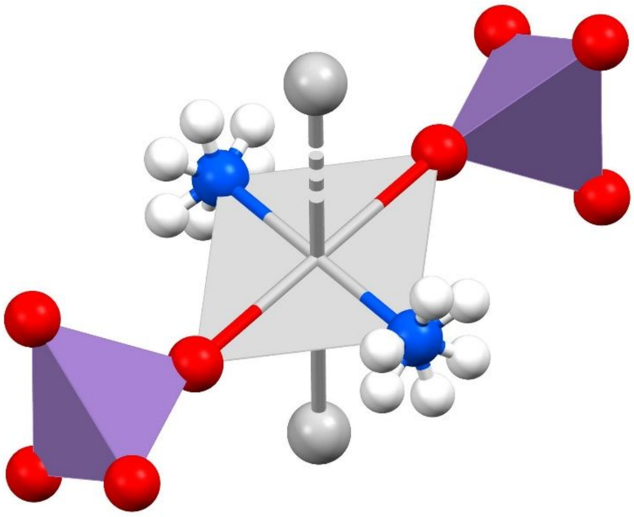

Figure S30. Octahedral coordination of the silver cations

The Ag-N and Ag-O distances are listed in ESI Table 2-4 for both polymorphs. All of the permanganate oxygens are involved in hydrogen bonds with the ammonia hydrogens. Hydrogen bonds for **LT-1** and **HT-1** are listed in ESI Table 3. In the solid phase of $[Ag(NH_3)_2]MnO_4$, an extensive hydrogen bonded network is formed with the participation of the ammonia molecules and the permanganate oxygens. The ammonia hydrogens are disordered over two positions, which are in fact two different hydrogen bonding positions with the permanganate anions (Scheme-white and light blue hydrogens). A certain flexibility of the ammonia positions may be deduced on the basis of the two different hydrogen positions, i.e., the ammonia position can be switched between the two hydrogen bonding sites.

Calculated powder diffraction patterns for of the two polymorphs are compared on Figure S31.

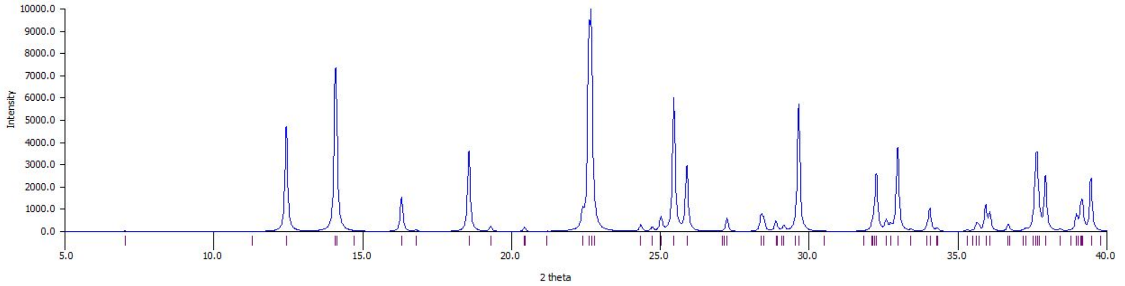

a

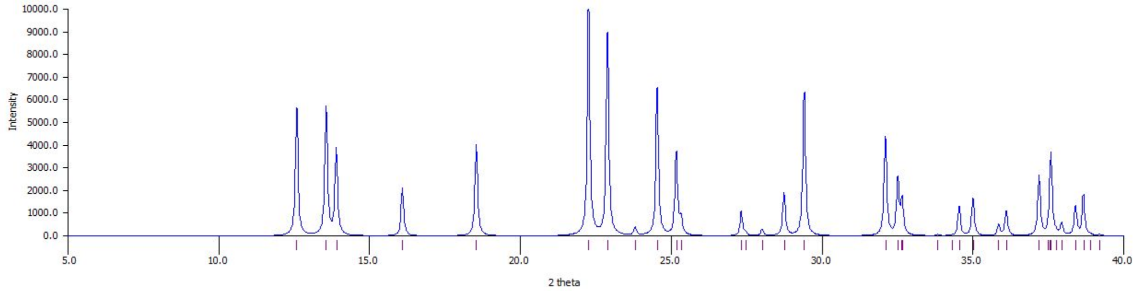

b

Figure S31. Generated powder diffraction patterns of **M-1** (a) and **M-2** (b) (CuK_{α} radiation)

Table S1. Crystal data and structure refinement for compounds **LT-1** and **HT-1**

Empirical formula	Ag1 H6 Mn1 N2 O4	Ag1 H6 Mn1 N2 O4
Formula weight	260.865	260.865
Temperature	100.0(1)	180.0(1)
Radiation and wavelength	Mo-Kα, λ =0.71073Å	Mo-Kα, λ =0.71073Å
Crystal system	monoclinic	monoclinic
Space group	*P* 2/m	*I* 2/m
Unit cell dimensions	*a* =7.9095(5)Å	*a* =7.8112(3)Å
	b =6.0205(4)Å	*b* =6.0682(2)Å
	c =12.6904(11)Å	*c* =13.1260(5)Å
	α =90°	α =90°
	β =98.056(7)°	β =96.438(4)°
	γ =90°	γ =90°
Volume	598.34(8)Å^3	618.25(4)Å^3
Z	4	8
Density (calculated)	2.896 Mg/m^3	2.803 Mg/m^3
Absorption coefficient, μ	5.317 mm^{-1}	5.145 mm^{-1}
F(000)	496	496
Crystal colour	colorless	colorless
Crystal description	prism	prism
Crystal size	0.200 x 0.150 x 0.150 mm	0.50 x 0.20 x 0.15 mm
Absorption correction	Multi-scan	Multi-scan
Max. and min. transmission	0.69743,1.00000	0.61697,1.00000
θ–range for data collection	3.243 ≤ θ ≤ 29.862°	2.899 ≤ θ ≤ 29.881°
Index ranges	-11 ≤ *h* ≤10;-8≤ *k* ≤8;-17 ≤ *l* ≤17	-10 ≤ *h* ≤10;-8≤ *k* ≤8;-16 ≤ *l* ≤18
Reflections collected	1673	6973
Completeness to 2θ	0.997	1.000
Independent reflections	1673 [*R*(int) =0.0466]	921 [*R*(int) =0.0279]
Reflections *I*>2σ(*I*)	1279	845
Refinement method	full-matrix least-squares on *F*2	full-matrix least-squares on *F*2

Data / restraints / parameters	1673 /0 /100	921 /0 /50
Goodness-of-fit on F^2	1.094	1.018
Final R indices [$I>2\sigma(I)$]	$R1$ =0.0356, $wR2$ =0.0807	$R1$ =0.0205, $wR2$ =0.0504
R indices (all data)	$R1$ =0.0519, $wR2$ =0.0886	$R1$ =0.0241, $wR2$ =0.0523
Max. and mean shift/esd	0.000;0.000	0.000;0.000
Largest diff. peak and hole	1.880;-0.978 e.Å^{-3}	1.541;-0.639 e.Å^{-3}

Table S2. Interatomic distances and angles in compounds **LT-1** and **HT-1**.

**LT-1**					
Ag1-N1	2.15(2) Å	Ag1-O4	2.69(2) Å	N1-Ag1-O4	89.4(8)°
Ag2-N2	2.15(2) Å	Ag2-O2	2.77(2) Å	N2-Ag2-O2	73.5(8)°
Ag3-N3	2.10(2) Å	Ag3-O5	3.04(2) Å	N3-Ag3-O5	85.9(8)°
Ag4-N4	2.12(2) Å	Ag4-O3	2.86(2) Å	N4-Ag4-O3	70.8(8)°
**HT-1**					
Ag1-N1	2.112(3) Å	Ag1-O2	2.685(2) Å	N1-Ag1-O2	81.2(1)°
Ag2-N2	2.113(3) Å	Ag2-O1	3.054(2) Å	N2-Ag2-O1	78.6(1)°

Table S3. Comparison of Ag-Ag and Ag-N bond distances (Å) or NAgN angles (°) in $[Ag(NH_3)_2]X$ compounds

X(-)	Temperature, K	d_{Ag-Ag}	d_{Ag-N}	N-Ag-N angles	Ref.
MnO_4, LT	100	3.010	2.100; 2.115; 2.142; 2.150	180	our work our work
MnO_4, HT	180	3.034	2.112; 2.113	180	
ClO_4, LT	170	3.020	2.115; 2.139	180	Nockemann, ZAAC
ClO_4, HT	293	3.089	2.129; 2.138; 2.145; 2.160	180	
SO_4, LT	298	3.200	2.110	174.3	Zachw. Z. krist.
NO_3	223	3.131	2.122; 2.125	180	Yamaguchi, Acta Chem: Scand.

Table S4. Analysis of Potential Hydrogen Bonds and Schemes with d(D...A) < R(D)+R(A)+0.50, d(H...A) < R(H)+R(A)-0.12 Ang., D-H...A > 100.0 Deg

**LT-1**					
Donor --- H....Acceptor	D - H	H...A	D...A	D - H...A	symm. op.
N1 --H1A ..O1	0.89	2.57	3.24(2)	132	x,y,z
N1 --H1A ..O5	0.89	2.46	3.18(3)	139	1-x,y,1-z
N1 --H1B ..O6	0.89	2.39	3.28(3)	172	-x,2-y,1-z
N1 --H1C ..O2	0.89	2.20	3.074(6)	169	x,1+y,z
N2 --H2A ..O2	0.89	2.54	2.98(3)	112	x,y,z
N2 --H2A ..O6	0.89	2.31	3.16(2)	162	-x,-1+y,1-z
N2 --H2B ..O4	0.89	2.53	3.119(8)	124	x,-1+y,z
N2 --H2C ..O4	0.89	2.26	3.119(8)	162	x,y,z
N3 --H3A ..O1	0.89	2.60	2.98(2)	107	-1+x,y,-1+z
N3 --H3A ..O1	0.89	2.14	2.98(2)	158	-1+x,2-y,-1+z
N3 --H3B ..O3	0.89	2.53	3.148(9)	127	-x,1+y,1-z
N3 --H3C ..O3	0.89	2.27	3.148(9)	169	-x,y,1-z
N4 --H4A ..O5	0.89	2.47	3.152(8)	134	x,y,z
N4 --H4A ..O1	0.89	2.40	3.11(2)	137	1-x,y,1-z
N4 --H4B ..O5	0.89	2.32	3.152(8)	156	x,-1+y,z
N4 --H4B ..O1	0.89	2.58	3.11(2)	119	1-x,1-y,1-z
N4 --H4C ..O6	0.89	2.55	3.11(2)	121	x,-1+y,z
N4 --H4C ..O3	0.89	2.38	2.94(3)	121	-x,y,1-z
N4 --H4C ..O6	0.89	2.38	3.11(2)	139	x,2-y,z
**HT-1**					
Donor --- H....Acceptor	D - H	H...A	D...A	D - H...A	symm. op.
N1 --H1B ..O1	0.89	2.48	3.2903(1)	152	3/2-x,-1/2+y,3/2-z
N1 --H1B ..O3	0.89	2.41	3.0680(1)	131	3/2-x,-1/2+y,3/2-z
N1 --H1C ..O3	0.89	2.39	3.2487(1)	162	x,y,z
N1 --H1C ..O3	0.89	2.55	3.0680(1)	118	3/2-x,3/2-y,3/2-z
N2 --H2A ..O3	0.89	2.15	3.0266(1)	170	x,1-y,z
N2 --H2B ..O3	0.89	2.51	3.0266(1)	118	x,-1+y,z
N2 --H2B ..O2	0.89	2.34	3.1651(1)	154	1-x,-1+y,1-z

Grinberg determined a parameter for ammine complexes based on the values of $\delta_s(NH_3)$ between the uncoordinated (gaseous) ammonia (968 cm^{-1}) with RBS=0 and the most stable ammine complex ($[Pt(NH_3)_4]Cl_2$) (1370 cm^{-1}) with RBS=100. The RBS_0 is defined as the lowest experimental found RBS among the diamminesilver complexes which is supposed to the value without contribution of the hydrogen bond interaction (RBS_0=53.0).

Table S5. RBS values for $[Ag(NH_3)_2]^+$ cations in compounds **LT-1** and **HT-1** at various temperatures

T, K (Compound)	RBS	RBS-RBS_0	H-bond contribution
87 K, **LT-1**	66.9	13.8	20.6
	64.5	11.4	17.8
	57.6	4.5	7.8
	56.2	3.1	5.7
	54.8	1.7	3.3
	53.1	0.1	0.2
180 K, **HT-1**	65.5	12.5	19.1
	56.9	3.9	6.9
	55.2	2.2	4.0
	53.1	0.1	0.2
300 K, **HT-1**	64.8	11.8	18.2
	55.5	2.5	4.5
